# Larval application of sodium channel homologous dsRNA restores pyrethroid insecticide susceptibility in a resistant adult mosquito population

**DOI:** 10.1186/s13071-016-1634-y

**Published:** 2016-07-14

**Authors:** Ana Caroline Dalla Bona, Rodrigo Faitta Chitolina, Marise Lopes Fermino, Lisiane de Castro Poncio, Avital Weiss, José Bento Pereira Lima, Nitzan Paldi, Emerson Soares Bernardes, Jonathan Henen, Eyal Maori

**Affiliations:** Forrest Brasil Tecnologia Ltda, Curitiba, PR Brazil; Forrest Innovations Ltd, Caesarea, Israel; Instituto Oswaldo Cruz - Fiocruz, Laboratório de Fisiologia e Controle de artrópodes vetores, Rio de Janeiro, RJ Brazil; Nuclear Energy Research Institute, Radiopharmacy Center, São Paulo, Brazil

**Keywords:** Insecticide resistance, Pyrethroids, Voltage-gated sodium channel, RNAi, *Aedes aegypti*, Mosquitoes

## Abstract

**Background:**

Mosquitoes host and pass on to humans a variety of disease-causing pathogens such as infectious viruses and other parasitic microorganisms. The emergence and spread of insecticide resistance is threatening the effectiveness of current control measures for common mosquito vector borne diseases, such as malaria, dengue and Zika. Therefore, the emerging resistance to the widely used pyrethroid insecticides is an alarming problem for public health. Herein we demonstrated the use of RNA interference (RNAi) to increase susceptibility of adult mosquitoes to a widely used pyrethroid insecticide.

**Methods:**

Experiments were performed on a field-collected pyrethroid resistant strain of *Ae. aegypti* (Rio de Janeiro; RJ). Larvae from the resistant *Ae. aegypti* population were soaked with double-stranded RNAs (dsRNAs) that correspond either to voltage-gate sodium channel (VGSC), P-glycoprotein, or P450 detoxification genes and reared to adulthood. Adult mortality rates in the presence of various Deltamethrin pyrethroid concentrations were used to assess mosquito insecticide susceptibility.

**Results:**

We characterized the RJ *Ae. aegypti* strain with regard to its level of resistance to a pyrethroid insecticide and found that it was approximately 6 times more resistant to Deltamethrin compared to the laboratory Rockefeller strain. The RJ strain displayed a higher frequency of Val1016Ile and Phe1534Cys substitutions of the VGSC gene. The resistant strain also displayed a higher basal expression level of VGSC compared to the Rockefeller strain. When dsRNA-treated mosquitoes were subjected to a standard pyrethroid contact bioassay, only dsRNA targeting VGSC increased the adult mortality of the pyrethroid resistant strain. The dsRNA treatment proved effective in increasing adult mosquito susceptibility over a range of pyrethroid concentrations and these results were associated with dsRNA-specific small interfering RNAs in treated adults, and the corresponding specific down regulation of VGSC gene expression level. Finally, we demonstrated that the efficiency of our approach was further improved by ‘tiling’ along the VGSC gene in order to identify the most potent dsRNA sequences.

**Conclusions:**

These results demonstrate that dsRNA applied to mosquito larvae retains its biological activity into adulthood. Thus, the RNAi system reported here could be a useful approach to control the widespread insecticide resistance in mosquitoes and other insect vectors of human diseases.

**Electronic supplementary material:**

The online version of this article (doi:10.1186/s13071-016-1634-y) contains supplementary material, which is available to authorized users.

## Background

Mosquitoes are responsible for a huge detrimental impact on global public health. They are by far the deadliest animals to humans on the planet [1]. Approximately 3.3 to 3.6 billion people are at risk of being infected by dengue or malaria, with around 21,000 dengue related deaths and 600,000 malaria related deaths annually [2]. Vector control still constitutes the critical element in the current global strategies for the control of vector-borne diseases such as malaria, Yellow fever, filariasis, Japanese encephalitis, West Nile fever, Rift Valley fever, Saint Louis encephalitis, chikungunya, dengue and Zika [3]. The Zika virus belongs to the family Flaviviridae and is transmitted to humans by *Aedes aegypti* mosquitoes [4]. Zika virus infection in pregnant women has been associated with various embryonic defects and abortion. Recently, two independent studies demonstrated that Zika causes placental damage as well as fetal intrauterine growth restriction, microcephaly and death in mice [5, 6]. The expanding *Aedes aegypti* spread and the emerging Zika virus disease are of worldwide concern.

For controlling the Zika and dengue vector *Aedes* (*Stegomyia*) *aegypti* (Linnaeus, 1762), several different approaches have been recently developed, such as the use of transgenic mosquitoes and *Wolbachia*-infected mosquitoes [7]. However, the traditional methods, i.e. environmental sanitation measures and chemical control are still the most widely used. For dengue control, efficient programs against *Ae. aegypti* were implemented in the late 1940s and early 1950s, resulting in the elimination of this species in almost all of the Americas, including Brazil [8]. Indoor residual spraying (IRS) and pyrethroid impregnated bed-nets (ITN) both targeting the *Anopheles* vector, led to unprecedented success in malaria prevention in Africa, preventing hundreds of millions of malaria cases over the last decade [9]. Although this chemical control has proven effective, it is fickle. The intensive use of insecticides and the constant exposure to them is resulting in strong selection pressure and development of resistance [10]. Indeed, recent monitoring of malaria vectors showed that resistance levels are increasing, 53 countries reported resistance to at least one insecticide, and 41 countries reported resistance to two or more insecticide classes [2, 11].

There are four main groups of neurotoxic insecticides registered for use for public health purposes, but pyrethroids are considered the most effective and safest insecticide class to combat the adult vectors [12, 13]. All pyrethroids act on the same target protein, the voltage-gated sodium channel (VGSC). This adulticide keeps VGSC neuron membranes open, stimulating the nerve cells to produce repetitive discharges which eventually cause paralysis and mosquito death [14]. To date, two main mechanisms are involved in pyrethroid resistance: enhanced metabolic detoxification and target site insensitivity [15, 16]. Indeed, single amino acid substitutions resulting from mutations in the VGSC gene have been associated with target site insensitivity to this class of insecticides. In natural populations of *Ae. aegypti* the most common mutations associated with resistance are the V1016G, F1534C [10, 17–19] and in *Anopheles* sp. is the L1014F [20, 21].

Among the new approaches implemented for pest control, one of the most promising is RNA interference (RNAi). RNAi is an evolutionarily conserved regulatory mechanism found in almost all eukaryotic organisms, which downregulates gene expression in a sequence-specific manner. RNAi is induced by the presence of double-stranded RNA (dsRNA) that is then processed by Dicer into small interfering RNAs (siRNAs). Later, the siRNAs recruit and guide the RNA-induced silencing complex (RISC) to the complementary RNA sequence, which is then cleaved and degraded [22–25]. The recruitment of this mechanism has led to the development of new methods for insect pest control, manipulation of insect disease vectors and disease management of beneficial insects [26–33]. Importantly, large-scale field implementation of this technology has proven to be successful in honey bees [34].

In mosquitoes, dsRNA delivered to larvae, resulted in increased toxicity of the larvicide temephos [35]. Additionally, dsRNA fed directly to adults achieved a gene knockdown effect at 48 h post-feeding [36]. However, these findings were focused on delivering dsRNA and assessing gene silencing and related-phenotype in the same mosquito developmental stage. Recent observations suggest that RNAi triggered in mosquito larvae could be maintained until adulthood [37, 38]. Nevertheless, these studies did not establish molecular evidence for a RNAi mechanism that persists across developmental stages in mosquitoes.

Herein we describe a novel RNAi-based approach for vector control. Through treating *Ae. aegypti* mosquito larvae with dsRNA targeting the VGSC gene, we were able to induce and characterize long-lasting gene silencing, which persisted into the mosquito adult. Moreover, the treatment successfully restored pyrethroid susceptibility in a field-collected resistant strain of *Ae. aegypti*. This study shows that dsRNA delivered during the mosquito larval stage can be potentially useful in integrated vector control programs, by helping to break down the resistance of adult mosquitoes to the still widely used pyrethroid insecticides.

## Methods

### Mosquito populations

*Ae. aegypti* eggs of the Rockefeller strain (Rock), Rio de Janeiro strain (RJ, Nova Iguaçu, 23°S, 43°W) were obtained from the Laboratório de Fisiologia e Controle de Artrópodes Vetores of the Instituto Oswaldo Cruz (Rio de Janeiro). Adult mosquitoes were maintained as described by Rutledge et al. [39], with modifications. Briefly, mosquitoes were fed on a 10 % sugar solution and were reared at 26–28 °C, 70–80 % relative humidity and a 12:12 photoperiod. Adult females were fed with artificial feeders, using natural pork gut membrane and defibrinated sheep blood (Laborclin®).

### DsRNA synthesis

Total cDNA from *Ae. aegypti* adults was employed as the template for synthesis of dsRNA sequences using specific primers containing T7 promoter sequences. The dsRNAs were synthetized by in vitro transcription using Megascript RNAi kit (Ambion) according to the manufacturer’s instructions. Integrity and purity of dsRNA were verified by non-denaturing 1 % agarose gel electrophoresis, and concentration was determined spectrophotometrically. We produced one dsRNA sequence targeting the *Ae. aegypti* CYP9J26 gene [NCBI: XM_001649047.2, VectorBase: AAEL014609-RA], one targeting the CYP9J32 gene [NCBI: XM_001653404.2, VectorBase: AAEL008846-RA], one targeting P-glycoprotein gene [NCBI: XM_001654442.1, VectorBase: AAEL010379-RA], and sequences targeting the VGSC gene [NCBI: KC107440.1, VectorBase: AAEL006019-RD]: the VGSC_422_ dsRNA and 8 different tiling sequences (Tile 1, Tile 2, Tile 3, Tile 4, Tile 5, Tile 6, Tile 7 and Tile 8). The primer sequences, as well as the position of each dsRNA in the VGSC gene are indicated in Additional file 1. Artificial dsRNA (Random-dsRNA) sequence, which has no perfect homology greater than 19 bp with the *Ae. aegypti* genome, was generated by “Random DNA Sequence Generator” (http://www.faculty.ucr.edu/~mmaduro/random.htm). The Random-dsRNA’s full sequence (357 bp excluding the T7 promoters) is shared in Additional file 2. Additional dsRNA (Random, VGSC_422_ and VGSC_T6_) was purchased from AgroRNA.

### DsRNA soaking assay

For each treatment, 100 third instar larvae were placed inside a 50 ml plastic cup with 50 μl of autoclaved food solution (6 g/100 ml – 40 % cat food, 40 % rabbit food, 10 % yeast, 10 % fish flakes) and the desired amount of indicated dsRNA ranging from 0.05 to 0.5 μg/μl in a total of 3 ml autoclaved water. The untreated group was kept in 3 ml autoclaved water as the control group. One hundred third instar larvae were soaked for 24 h and after that transferred to a new tray with 1 l of aged water. Larvae were allowed to develop until they became adults. All procedures and larval development were performed at 26–28 °C. Adult females were used in the adulticide bioassays as described below.

### Pyrethroid bioassays

Deltamethrin (PESTANAL®; Sigma-Aldrich) diluted in acetone was used in the adulticide bioassays, performed as previously described [40]. Bottles with acetone only served as a control. The resistance status of the RJ population (without dsRNA treatment of larvae) was measured and compared to the Rock strain using qualitative assays (diagnostic dose: 2 μg/ml; diagnostic time: 30 min). The knockdown effect was evaluated after 24 h into the pyrethroid bioassay. Populations with a mortality rate above 98 % were considered susceptible; whereas populations with mortality rates below 80 % were considered resistant. Populations with mortality rates between 80 and 98 % were considered populations with modifications in susceptibility [41].

For the quantitative bioassays, female adults (with dsRNA treatment of larval stage) were exposed for 30 min at different deltamethrin concentrations, ranging from 0 to 2 μg/ml, or at the indicated concentration. Three to four bottles (250 ml) were impregnated with each insecticide concentration. For each bottle, 10 to 15 unfed females (5–7 day-old maximum) were used. Females (live and dead) were immediately collected following the bioassays. After flash freezing, samples were stored at -80 °C for analysis of gene silencing.

### Mating assay and F1 generation effect

Third instar larvae were treated with VGSC dsRNA or left untreated and grown until the pupal stage where the pupae were separated by sex within each treatment. The sex separation was performed at the pupal stage in order to ensure that all the females remain virgin. After their emergence, the adults were crossed as follows: X1 males untreated (water) crossed with females untreated (water); X2 males treated with dsRNA VGSC crossed with females treated with dsRNA VGSC; X3 males treated with dsRNA VGSC crossed with females untreated (water); X4 males untreated (water) crossed with females treated with dsRNA VGSC. After mating, the females within each crossing were blood fed and allowed to lay their eggs. These eggs (F1 generation) were grown until the adult stage and then were used for the pyrethroid bioassay.

### Genotyping assays

Individual mosquitoes from either RJ or Rock strain were genotyped at position 1016 and 1534 of the genomic DNA using allele-specific PCR (AS-PCR). Following DNA extraction, PCR reactions were performed using GoTaq® Green Mastermix (Promega Corporation), 100 ng genomic DNA, 0.24 μM of the reverse primer and 0.12 μM of each one of the forward primer with a final volume of 12.5 μl. The amplification reactions were performed according to reference [42]. The sequences of primers used were described by Linss et al. [10] and are shown in Additional file 3.

The amplified alleles were analyzed on a 10 % polyacrylamide gel. For the mutation Val1016Ile, a 78 bp band refers to the mutant allele (1016Ile) and a band with 98 bp represents the wild type allele (1016Val). For the mutation Phe1534Cys, the 110 bp band is in linkage with the mutant allele (1534Cys) and the 90 bp band is linked to the wild type allele (1534Phe).

### Reverse transcription and relative mRNA quantification

VGSC expression levels were measured using Real time PCR following the 2^-ΔΔCT^ method [43]. Total RNA was extracted using TRIzol reagent (Invitrogen Life Technologies, Carlsbad, CA, USA), according to the manufacturer’s instructions. cDNA synthesis was performed in a final volume of 20 μl, using ImProm-II Reverse Transcriptase (Promega Corporation, Madison, WI, USA). The reaction mixture contained 2 μg of total RNA, 20 pmol of oligo dT primer (Invitrogen Life Technologies, Carlsbad, CA, USA), 40 U of RNAsin, 1 mM of dNTP mix, and 1 U of reverse transcriptase. PCR amplification and analysis were achieved using an ABI Prism 7500 sequence detector (Applied Biosystems, Foster City, CA, USA). All reactions were performed with SYBR Green PCR Master Mix (Applied Biosystems) using a 10 μl final volume in each reaction, which contained 1 μl of template cDNA, 2.5 pmol of each primer and 5 μl of SYBR Green. The cycles were processed according to the manufacturer’s instructions. Each sample was tested in duplicate and all quantifications were normalized using α-tubulin as the endogenous control. The primers used for all PCR amplifications are described in Additional file 1.

Alternatively, mRNA quantification was determined using the QuantiGene Plex 2.0 branched DNA assay. Briefly, total RNA from each sample (400 ng) was analyzed by using a Bio-Plex 200 system array reader with Luminex 100 X-MAP technology, and data was acquired and analyzed by using Bio-Plex Data Manager 5.0 software (Bio-Rad Laboratories, Hercules, CA). Individual bead-based probe sets specific to the VGSC gene and the endogenous control gene S7 were designed by Affymetrix and are available in the company’s website. Assays were performed as recommended by the manufacturer. Data are shown as relative light units specific to mRNA expression, with normalization to levels of the endogenous control gene.

### Small RNA sequencing

Total RNA was extracted from a pool of 5 mosquitoes treated with Random or VGSC_422_ dsRNAs, using the Direct-zol™ RNA MiniPrep (Zymo Research), following the manufacturer’s instructions. RNA quality was verified using Agilent Total RNA Nano chip on Bioanalyzer 2100 instrument, and the quantification of total RNA for small RNA library preparation was performed using Qubit RNA BR Assay fluorimetric method (Invitrogen, USA).

One microgram (μg) RNA was used to generate a small RNA sequencing library using the TruSeq Small RNA Sample Prep Kit 7A (Illumina, USA), according to the manufacturer’s instructions. Briefly, T4 RNA ligase and T4 RNA ligase 2 Deletion mutant (Epicentre part #LR2D1132K, USA) were used to ligate RA5 and RA3 oligonucleotides to the 5′ and 3′ ends of the RNA, respectively. Adapter-ligated RNA was reverse-transcribed using a RTP primer and the resulting cDNA was amplified in an 11-cycle PCR that used RP1 and indexed RP1 primers. PCR products of 140–160 bp were isolated following electrophoresis through a 4 % agarose gel (Promega). Quality of the generated small RNA sequencing library was confirmed using Agilent High Sensitivity D1000 Screen Tape on TapeStation 2200 instrument. Sequencing was performed in a Hiseq 2500 (Illumina, USA) instrument, in a 100 bp single-end read run using HiSeq SBS clustering and sequencing kit v3-HS. Base-calling and generation of raw, de-multiplexed sequencing data in FASTQ format, was performed using BaseSpace (Illumina, USA) platform.

### Processing and analyses of RNA sequencing data

#### Mapping

Illumina Truseq small RNA 3′ adapters were trimmed using cutadapt [44] version 1.2.1. Untrimmed reads were discarded from the analysis. RNA-seq data were aligned to a broad institute assembly of *Ae. aegypti* genome [45] using STAR, an RNA-seq specific aligner, version 2.4.2a [46]; parameters were set for small RNA’s alignment: _outFilterMismatchNoverLmax = 0.05, outFilterMatchNmin = 16,_outFilterScoreMinOverLread = 0, outFilterMatchNminOverLread = 0, alignIntronMax = 1. Reads that were uniquely mapped to the VGSC gene were subjected to further downstream analysis.

#### Counting and normalization

The number of reads associated with each position along the silencing region and along the entire sodium channel mRNA were counted using coverageBed (bedtools version 2.17.0). Raw read counts were normalized to one million mapped reads per sample (i.e. RPM).

### Statistical analysis

Statistical analysis was performed with GraphPad prism 6.0 (GraphPad Software Inc,San Diego, CA). The corresponding tests performed are indicated in the legend of each figure.

## Results

### Characterization of pyrethroid resistance based on biological assays and molecular analysis of mutations in the VGSC

**Fig. 1:**
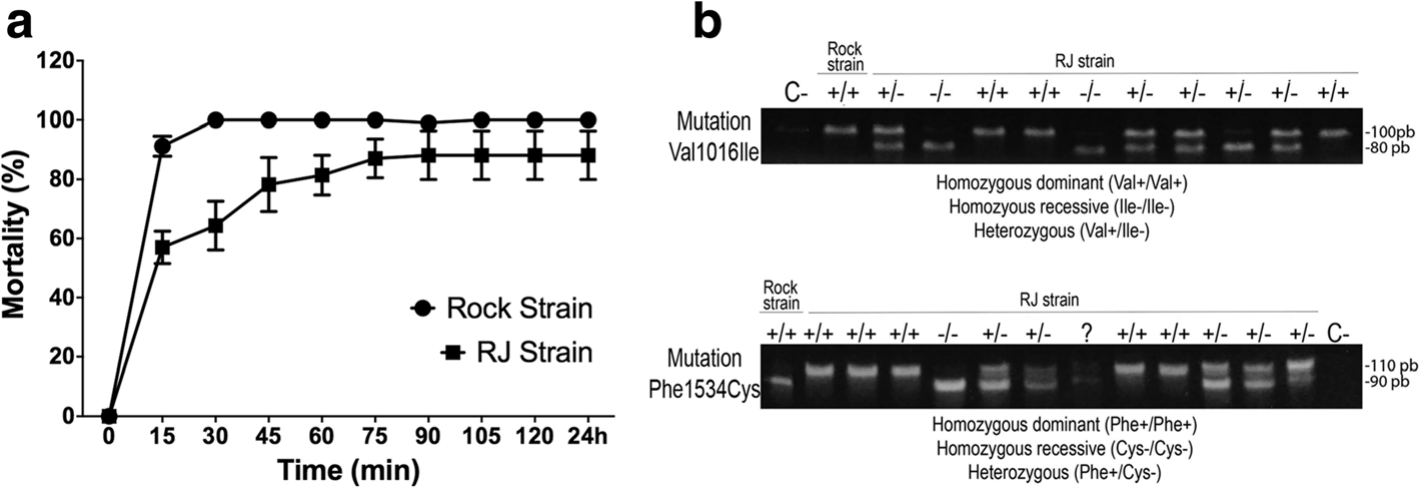
Characterization of pyrethroid resistance of two *Ae. aegypti* strains. **a** Five day-old *Ae. aegypti* female mosquitoes of the insecticide-susceptible ‘Rockefeller’ strain (Rock) and of the insecticide-resistant ‘Rio de Janeiro’ strain (RJ) were exposed to deltamethrin for up to 24 h and the percentage of mortality is shown. Data represent mean values of three experiments performed in triplicate ± standard deviation (SD). **b** Allele specific PCRs for genotyping *kdr* mutations were performed on adult *Ae. aegypti* mosquitoes from the RJ strain. Upper and lower panels represent reactions for the 1016 and 1534 mutation sites, respectively. In the upper panel, amplicons of approximately 80 and 100 bp correspond to alleles 1016 Val^+^ and 1016 Ile^*-*^, respectively. In the lower panel, amplicons of 90 and 110 bp correspond to alleles 1534 Phe^+^ and 1534 Cys^*-*^, respectively. Mosquitoes of the Rockefeller strain were used as positive homozygous dominant control for both mutation sites. C-: no template negative control

In order to check the resistance status of the RJ population, we performed a pyrethroid qualitative bioassay and found that the RJ population showed pyrethroid-induced mortality with a mean of 64 % at the diagnostic dose and time (2 μg/ml; 30 min), compared to the susceptible Rock strain, in which 100 % of the mosquitoes were already dead (Fig. 1a). This classifies the population as resistant to pyrethroids according to the WHO guidelines [41]. Moreover, we characterized the population at a molecular level through an AS-PCR which revealed that for the RJ population both mutations that are associated with pyrethroid resistance [10], Val1016Ile and Phe1534Cys, exist within the population (Fig. 1b). For the mutation Val1016Ile 45 individuals were analyzed and the resistance genotype Ile/Ile is present in 49 % of the population, reaching an allelic frequency of *f(Ile) =* 0.59. With the second mutation evaluated, Phe1534Cys 40 individuals were analyzed and 60 % of the population is characterized as having a resistance genotype Cys/Cys (*n* = 40 individuals) reaching a mutant allelic frequency of 77 % (*f(Ile) =* 0.77). The Rock strain proved to have a dominant homozygous genotype for both mutations (Additional file 4).

Based on WHO guidelines, the biological assay and the molecular analysis classifies the RJ field population as resistant to pyrethroid, whereas the Rock population is susceptible.

### Soaking larvae with specific VGSC dsRNA results in gene silencing and increased pyrethroid susceptibility

**Fig. 2:**
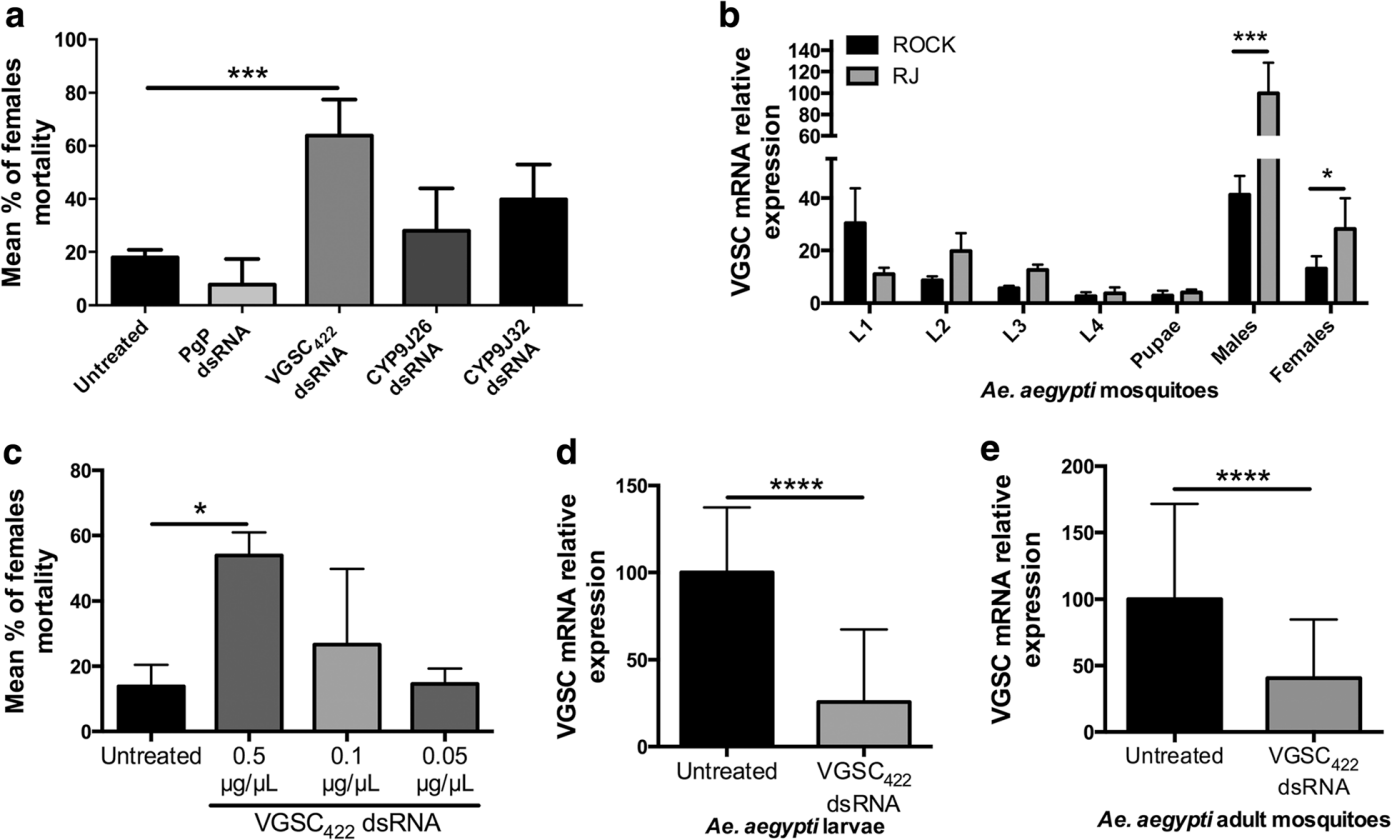
Silencing of the *Ae. aegypti* (RJ strain) VGSC results in increased susceptibility to a pyrethroid insecticide. **a** Mortality percentage in *Ae. aegypti* adults from RJ strain (after dsRNA treatment in larval stage) after 30 min of exposure to deltamethrin. Third-instar larvae were soaked in water (untreated) or in dsRNA corresponding to the VGSC (VGSC_422_ dsRNA), P-glycoprotein (PgP dsRNA) and P450 enzymes (CYP9J26, CYP9J32). Data are shown as the mean ± standard deviation of four replicates. Statistics: One-way Anova, followed by Tukey’s multiple comparisons test. **b** Comparative VGSC gene expression analysis between different developmental stages of Rock and RJ strains. Data are shown as the mean ± SD of 6 replicates. Statistics: Multiple *t*-test. **c** *Ae. aegypti* RJ strain larvae were soaked as described in **a**, but using three different concentrations of VGSC_422_, as indicated. Statistics: One-way Anova followed by Tukey’s multiple comparison test. **d** VGSC gene expression level was determined by quantitative RT-PCR (qRT-PCR) and performed on untreated or dsRNA-VGSC_422_ treated (0.5 μg/μL) *Ae. aegypti* RJ 4^th^ instar larvae. Data are shown as the mean ± SD of 6 experiments performed in triplicate. Statistics: *t*-test. **e** VGSC gene expression level was determined by qRT-PCR and performed on untreated or dsRNA-VGSC_422_ treated (0.5 μg/μl) *Ae. aegypti* RJ adults. Data are shown as the mean ± SD of 8 experiments performed in triplicate. Statistics: *t*-test. *P < 0.01, ***P < 0.0001; ****P < 0.00001

Pyrethroid resistance has been related to both metabolic and target site resistance mechanisms [18, 47]. Therefore, with the aim of increasing pyrethroid toxicity, we screened four different dsRNA molecules, targeting genes associated with insecticide detoxification or target site insensitivity, including: a) *P-glycoprotein* gene (PgP), which is related to a transmembrane protein of xenobiotic efflux [35]; b) ’*CYP9J26’* and c) ‘*CYP9J32’*, both associated with insecticide detoxification through the cytochrome P450 pathway [48] and d) *VGS*C, that codes the transmembrane protein which harbors the target site of the pyrethroid adulticides [49]. After the dsRNA treatment followed by the pyrethroid bioassay, we found that the VGSC_422_ dsRNA treatment increased the mortality of the RJ strain by about 50 % compared to untreated mosquitoes (Fig. 2a). The other treatments did not have any significant effect on mosquito sensitivity to the pyrethroid although dsRNAs corresponding to Pgp and CYP9J26 triggered gene silencing in the adult stage (Additional file 5). As the VGSC_422_ dsRNA is homologous to the VGSC gene, we checked the VGSC mRNA expression level in both resistant and susceptible populations in all developmental stages. We found that the RJ population has an increased expression of this gene in the adult stage, compared to the Rock population (Fig. 2b). Due to the high dose of dsRNA used to treat the larvae (0.5 μg/μl), we repeated the soaking experiment with decreased dsRNA amounts. This was done to check if the same phenotype of increased pyrethroid-driven mortality would be consistent at lower dsRNA concentrations (0.1 and 0.05 μg/μl), but no statistically significant phenotypic effect was observed (Fig. 2c). As only 0.5 μg/μl VGSC_422_ dsRNA showed a phenotype, we assayed its target gene expression in dsRNA-treated larvae and adults. We found gene silencing of the VGSC not only in the treated larvae, but also in adults (Fig. 2d and e).

**Fig. 3:**
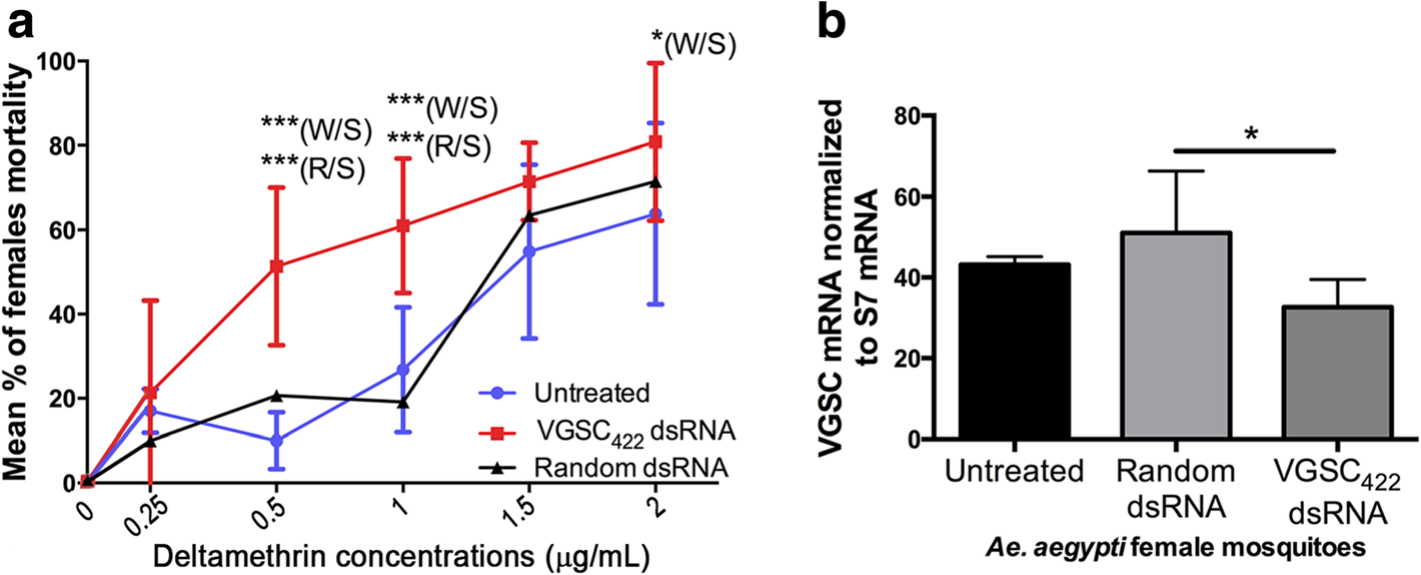
Soaking *Ae. aegypti* larvae with dsRNA-VGSC_422_ increases adult susceptibility to different concentrations of Deltamethrin. **a** Mortality percentage in 5 day-old *Ae. aegypti* adults from RJ strain (after dsRNA treatment in 3rd-instar larval stage - 0.5 μg/μl) at different Deltamethrin pyrethroid concentrations, after 30 min exposure. Data are shown as the mean ± standard deviation of four replicates, and 3 independent experiments were performed. Statistics: Two-way Anova followed by Bonferroni multiple comparison test. **b** Five day-old adult females (three replicates with 5 mosquitoes each) previously soaked with VGSC_422_ dsRNA, Random dsRNA or only water (untreated) were analyzed for VGSC mRNA expression levels using the Quantigene method. Final dsRNA soaking concentration: 0.5 μg/μl. Statistics: One-way Anova followed by Tukey’s multiple comparisons test. *P < 0.01, ***P < 0.0001. W: water (untreated); R: dsRNA random-treated; S: dsRNA VGSC_422_-treated

To further check efficacy and specificity of the VGSC_422_ dsRNA, we then performed a bioassay at different pyrethroid concentrations. VGSC_422_ dsRNA showed increased mortality of 15–35 % in the adults, compared with both untreated (water) and random dsRNA-treated mosquitoes (dsRNA with no homology to the mosquito genome). This difference in mortality rate is statistically significant at 0.5, 1.0 and 2.0 μg/ml of deltamethrin-impregnated bottles (Fig. 3a). Again, this consistent biological effect of increased mortality was linked to VGSC gene silencing assayed in adult mosquitoes and demonstrated by the Quantigene analysis; VGSC_422_ dsRNA applied to the larvae resulted in specific down regulation of the VGSC gene transcript in adult mosquitoes compared to a random dsRNA sequence, proving a specific action of the VGSC_422_ dsRNA molecule (Fig. 3b).

### Adult mosquitoes treated with VGSC_422_-dsRNA during the larval stage carry a siRNA population that corresponds only to the dsRNA target sequence

**Fig. 4:**
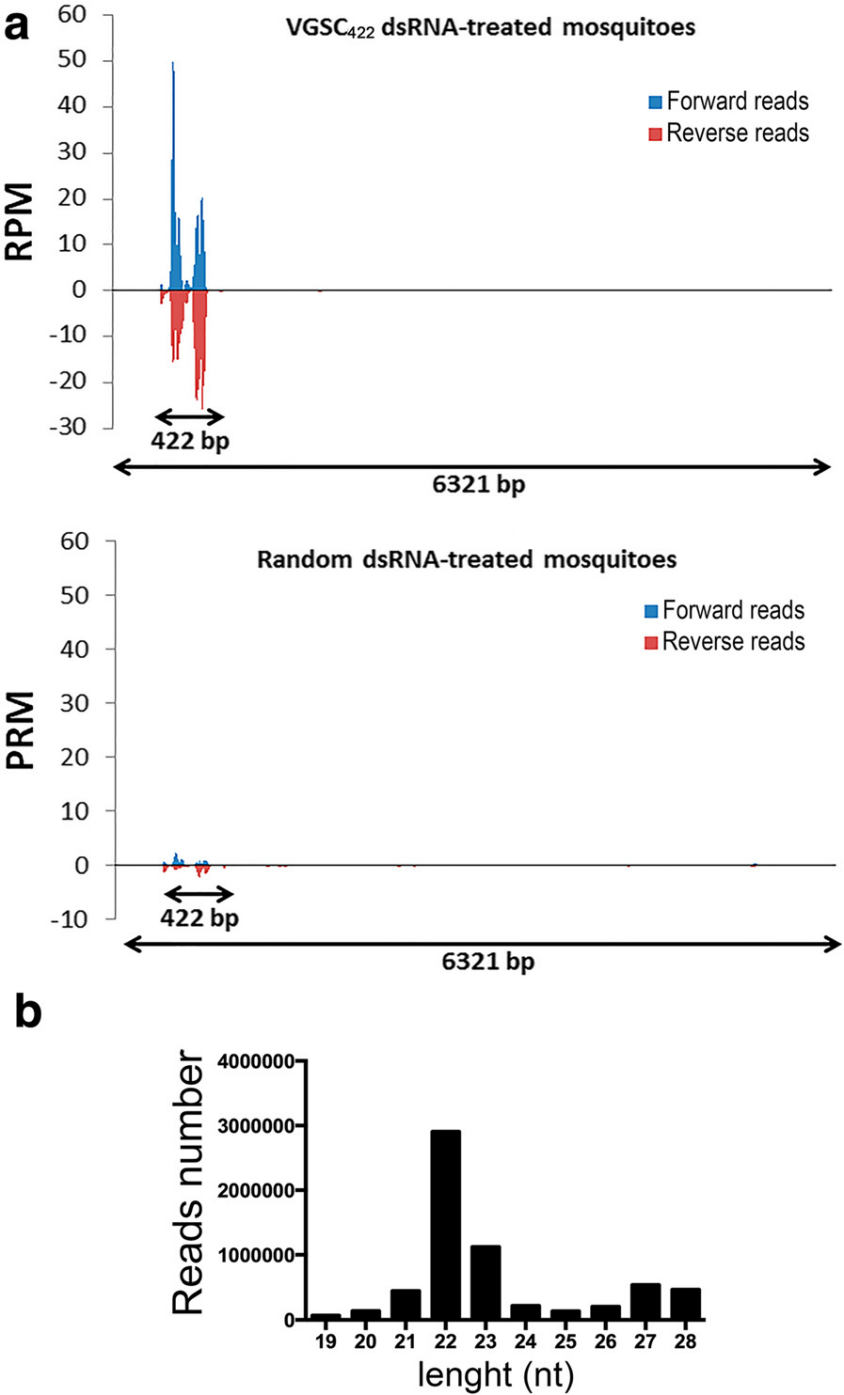
VGSC small RNAs persist until the adult stage of *Ae. aegypti* treated by dsRNA-VGSC during larval stage. **a** Small RNA libraries were constructed from 5 day-old adult mosquitoes previously soaked in VGSC_422_ dsRNA or Random dsRNA (0.5 μg/μl) and sequenced using Hiseq 2500. Data are presented as the number of reads normalized to one million mapped reads per sample (RPM). Forward strand (sense) reads are positive RPM values (depicted in *blue*); reverse strand (anti-sense) reads are in negative RPM values (depicted in *red*). Positions for which RPM values are > = 10 RPM were considered expressed. **b** Size distribution of small RNAs matching the VGSC422 transcript (Accession no. KC107440.1). nt: nucleotides

In order to identify the presence of VGSC-specific siRNAs in adult mosquitoes previously soaked in VGSC_422_ dsRNA, we prepared and sequenced small-RNA libraries from a pool of individuals treated by VGSC_422_-dsRNA or random-dsRNA. An average of 18,059,680 single-end 100-bp reads were generated per sample, and after performing the quality filter, the small-RNA sequence reads were aligned to the *Ae. aegypti* genome. The analysis of small-libraries constructed from VGSC_422_ dsRNA-treated mosquitoes revealed up to a 50.6-fold increase of small-RNA reads matching the 3′-position of the positive- and negative-sense RNA strands inside the VGSC full mRNA region compared with the Random dsRNA-treated mosquitoes, which corresponds to the 422 bp VGSC_422_ dsRNA homologous site (Fig. 4a). Importantly, no siRNAs were found outside of the dsRNA-homologous region. The non-appearance of secondary siRNAs indicates that transitive RNAi does not occur in mosquitoes. This observation is consistent with the absence of RNA-dependent RNA polymerases in mosquitoes [50, 51]. We also analyzed the size distribution of small RNAs inside the dsRNA-homologous region. As depicted in Fig. 4b, these small RNAs comprised an enriched population of 22 nucleotide length perfectly matching the dsRNA.

### Targeting different sequences (‘tiling’) along the sodium channel gene generated a refined dsRNA with a greater pyrethroid efficacy

**Fig. 5:**
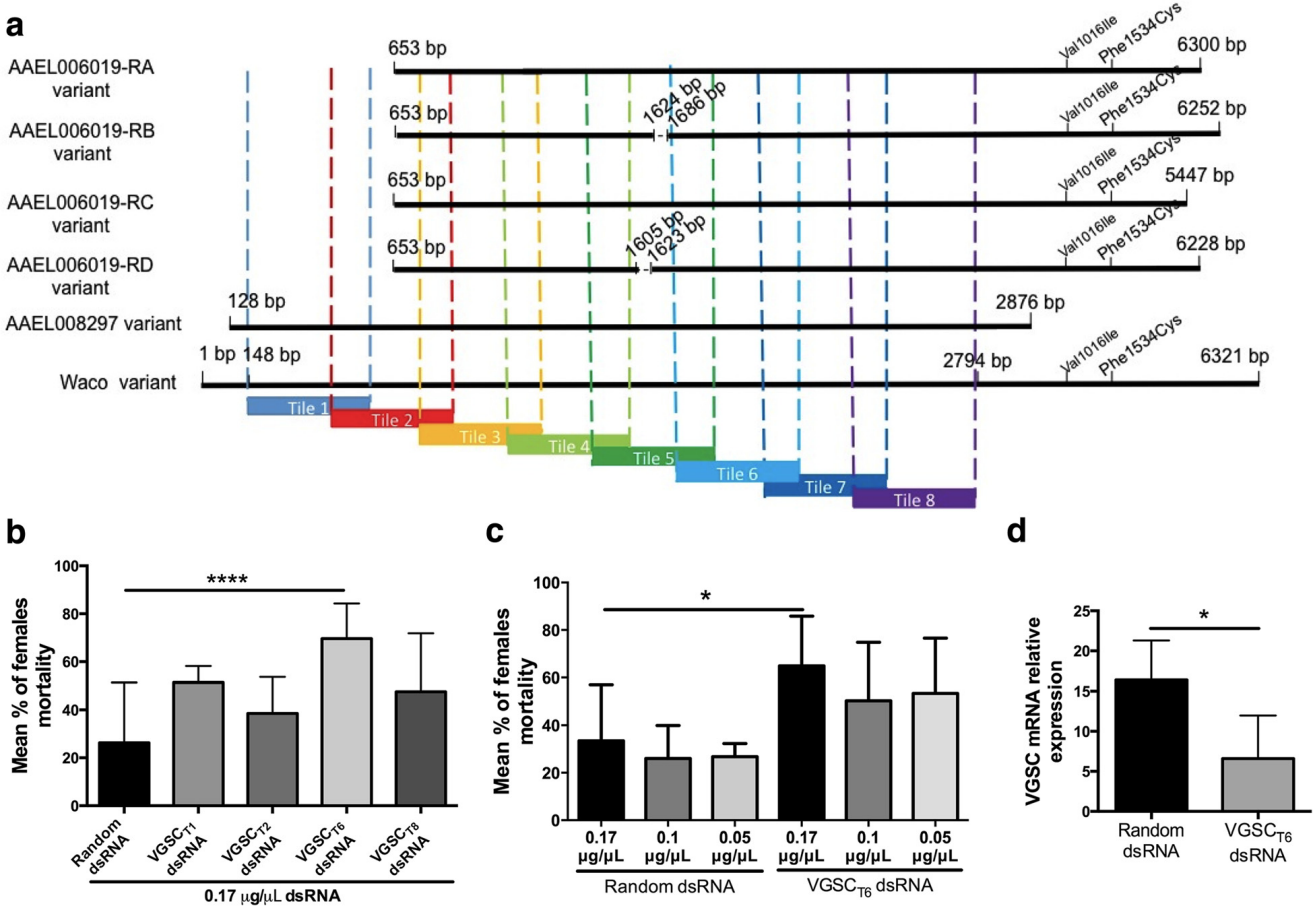
RNAi-effect optimization by refining the dsRNA constructs for the VGSC gene. **a** Schematic representation of 6 transcript variants of VGSC gene and the 8 overlapping dsRNA sequences used to silence the gene (Tile 1 to Tile 8). Deletion sites in variant RB and RD of variant AAEL006019 are indicated by a dotted line, as well as the position of mutation Val1016Ile and Phe1534Cys sites. **b** Mortality percentage of 5 day-old *Ae. aegypti* RJ adult females after 30 min exposure to deltamethrin. The adult females were treated at the 3rd larval stage by different dsRNA-VGSC tiles. Data show the mean ± standard deviation of three replicates from 3 independent experiments performed. Statistics: One-way Anova followed by Tukey’s multiple comparisons test. **c** Mortality percentage of 5 day-old *Ae. aegypti* RJ adult females after 30 min exposure to deltamethrin. The adult females were treated at the 3rd larval stage by three different concentrations of Random or VGSC_T6_ dsRNAs. Data are shown as the mean ± standard deviation of three replicates (2 independent experiments were performed). Statistics: One-way Anova followed by Tukey’s multiple comparisons test. **d** VGSC gene expression level was determined by qRT-PCR and performed on dsRNA-Random or dsRNA-VGSC_422_ treated and pyrethroid-affected *Ae. aegypti* RJ adults. Statistics: *t*-test *P < 0.05, ****P < 0.00001

After proving that the VGSC_422_ dsRNA treatment to larvae results in greater sensitivity to the pyrethroid adulticide through specific gene silencing, we directed our efforts to try to detect a dsRNA sequence that would result in an improved mortality phenotype. This would manifest as either increased mortality at the concentration previously used, or by reduction of the dsRNA concentration required to achieve a similar effect. To do so, we produced eight different but overlapping dsRNAs corresponding to the VGSC gene, named ‘Tile 1’ through ‘Tile 8’. The dsRNA tiles covered approximately half of the entire VGSC gene, and six of them are homologous to all the six transcript variants of VGSC annotated in this work and others (Fig. 5a, Additional file 6). Each tile was then used for the soaking procedure at a final concentration of 0.17 μg/μl, three times lower than the standard assay, and the females that emerged were subjected to the pyrethroid bioassay. Each tile sequence had a different effect on total mortality (Additional file 7). Four tiles out of eight that induced higher mortality rates in the initial screen were selected and compared to mosquitoes treated with Random dsRNA (Fig. 5b). From these four different sequences, the ‘Tile 6’ (VGSC_T6_) proved to be the most potent dsRNA due to the increased mortality rate and the concentration used compared with the random control (Fig. 5c) and the VGSC_422_ dsRNA (Fig. 2c).

**Fig. 6:**
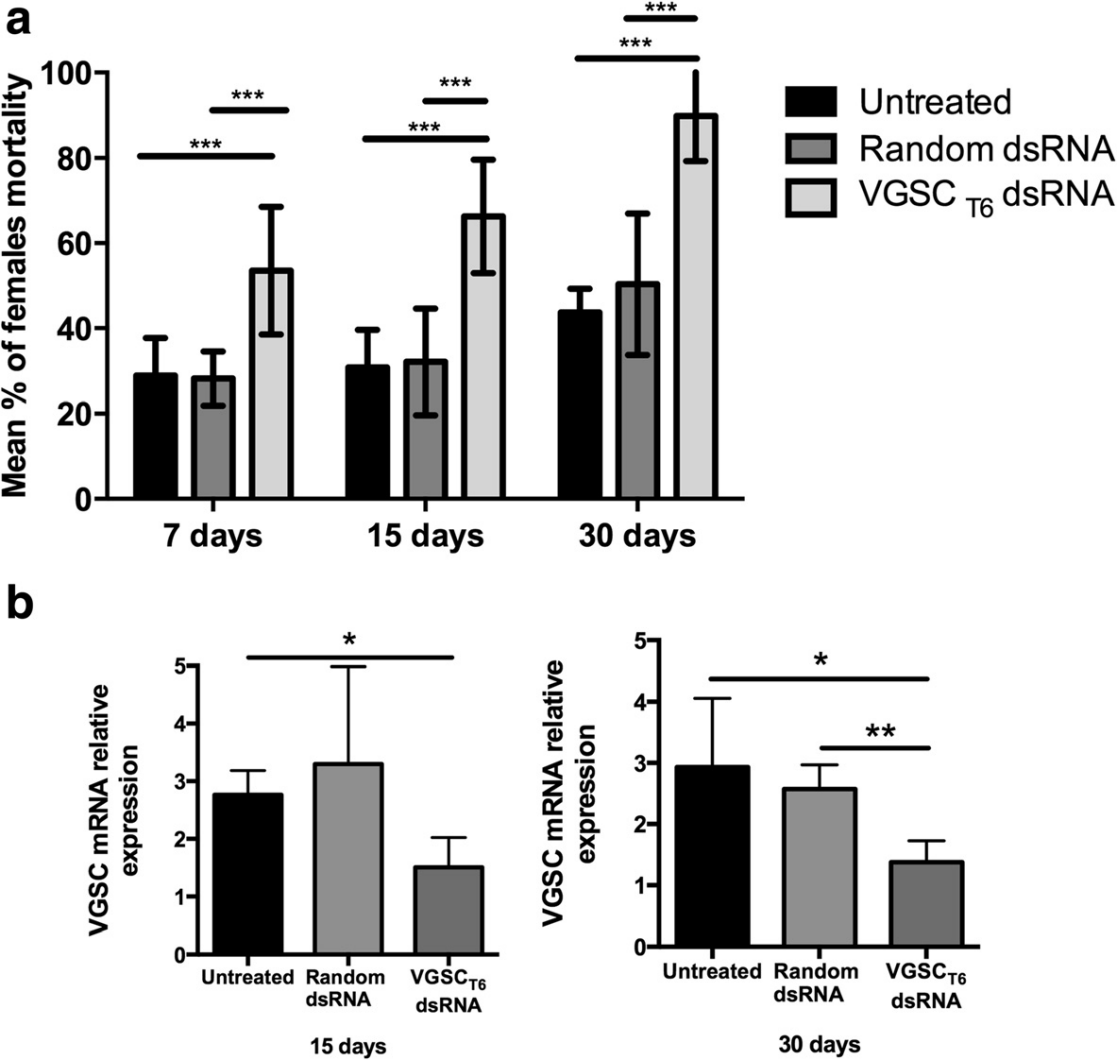
Soaking *Ae. aegypti* (RJ strain) larvae with dsRNA-VGSC_T6_ increases pyrethroid (deltamethrin) susceptibility in adult mosquitoes of different ages. **a** Mortality percentage of 7, 15 and 30 day-old adult *Ae. aegypti* females from the RJ strain after 30 min exposure to deltamethrin pyrethroid (5 μg/ml). Adults were soaked in 0.5 μg/μl dsRNA in their previous 3rd instar larvae stage. Data represent the mean ± standard deviation of four treatment replicates, and 3 independent experiments were performed. Statistics: One-way Anova followed by Tukey’s multiple comparison test. **b** Adult females (three biological replicates with 5 mosquitoes each) previously soaked (0.5 μg/μl) with VGSC_T6_ dsRNA, Random dsRNA or only water (untreated) were analyzed by qRT-PCR for VGSC mRNA expression levels. Statistics: One-way Anova followed by Tukey’s multiple comparisons test. *P < 0.05; **P < 0.01; ***P < 0.0005

As a clear biological effect resulting from the 3-fold lower concentration of dsRNA treatment was identified in T6, we showed that VGSC gene silencing was being induced. qRT-PCR analysis revealed a statistically significant 2.5 fold down regulation of the VGSC gene in adult mosquitoes treated with VGSC_T6_ dsRNA compared to Random dsRNA treated mosquitoes (Fig. 5d). With a refined RNAi trigger, we further investigated the VGSC_T6_ dsRNA effect at different adult ages with regards to pyrethroid sensitivity. Following larval VGSC_T6_ dsRNA application, significant and increasing pyrethroid susceptibility was detected in 7, 15 and 30 day-old adults. This cross-developmental RNAi effect was supported by stable VGSC gene knockdown (Fig. 6). Interestingly, in the absence of pyrethroid, the VGSC_T6_ dsRNA uptake did not affect vitality and behavior of treated larvae and adults from both Rock and RJ strains (Additional file 8). Hence, we conclude that the phenotypic impact of the VGSC_T6_ dsRNA is specific and sequence dependent.

## Discussion

The emergence and geographical expansion of pyrethroid resistant mosquito populations is a great concern to vector-borne disease control measures. In the present study we report on a gene silencing approach that restores pyrethroid susceptibility of resistant *Ae. aegypti* mosquitoes. Specific dsRNA application to larvae downregulated the corresponding sodium channel gene in both larval and adult stages. This sustainable larva-to-adult silencing was sufficient to significantly reduce pyrethroid insensitivity of adults, demonstrating that reversal of insecticide resistance is a promising approach for pest control.

Resistance to a pyrethroid insecticide could evolve through various mechanisms including reduced uptake (behavioral resistance), enhanced metabolic detoxification or via gene modification that affects the insecticide’s affinity to- or activity on its target site [12, 52]. Indeed, genotyping a wild resistant strain (RJ) revealed two mutations in the VGSC gene that have been associated with increased resistance to pyrethroids (Fig. 1b) [10, 18]. Although our RNAi screen also included detoxification components, P450 and PgP, a significant phenotypic effect was observed only with dsRNA that targets the VGSC. This does not exclude the involvement of metabolic detoxification in our wild pyrethroid resistant strain. However, it further supports that the presence of mutated VGSC is involved in pyrethroid resistance in our field population.

**Fig. 7:**
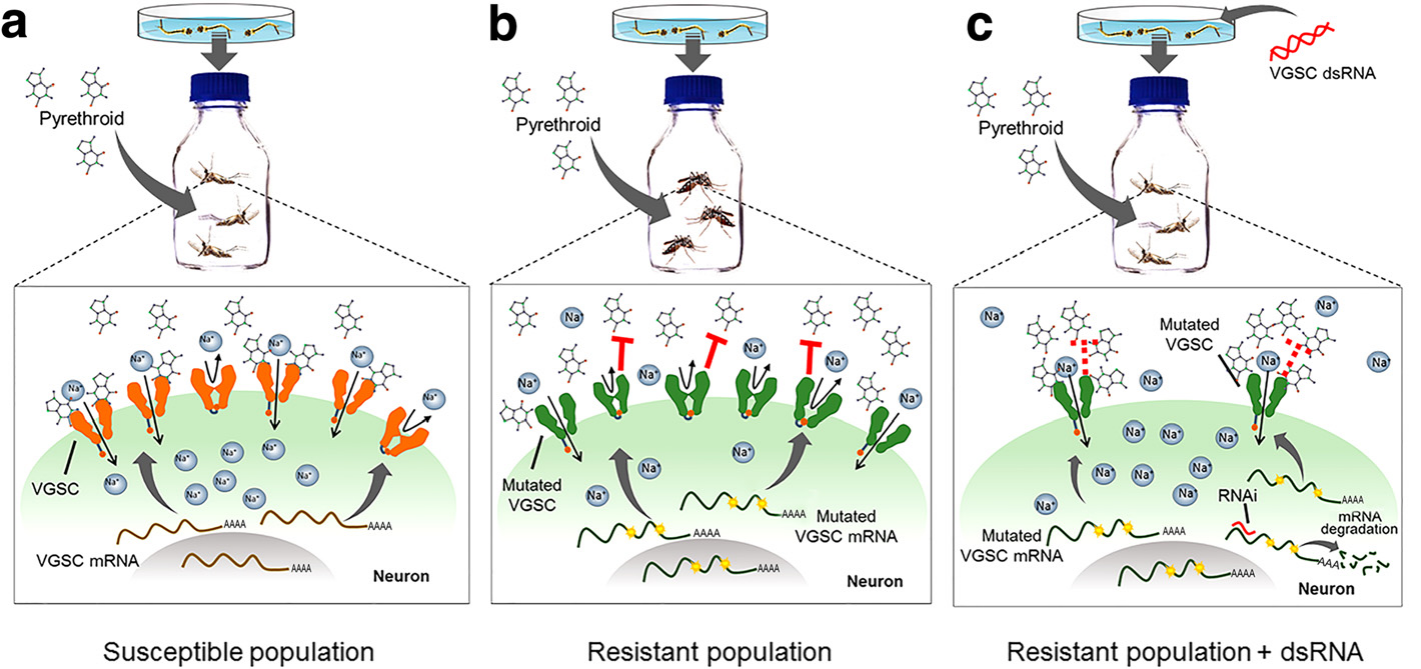
A proposed RNAi mode of action that impairs pyrethroid resistance. **a** Susceptible mosquitoes do not carry mutations that affect pyrethroid activity on the target VGSC (wild type VGSC in *orange*). The pyrethroid molecules bind to the VGSC and promote a constantly open state of the VGSC. This results in an increased influx of Na^+^ ions into the cell, causing repetitive impulses, exhaustion and eventually death. **b** Resistant mosquitoes carry mutated modified VGSC (mVGSC in *green*) with reduced interaction with pyrethroids (*red* colored “T”). Therefore, the toxic consequence of the insecticide is nullified when resistant mosquitoes are exposed to the pyrethroid concentration that effects the susceptible population. **c** RNAi decreases the mVGSC density and therefore the insecticide:target ratio is shifted when mosquitoes are exposed to pyrethroid concentrations that have no effect on resistant populations. This ratio alteration represents an increased pyrethroid molecule number per target. The elevated insecticide concentration makes up for its impaired interaction with the mVGSC target, leading to binding and the associated harmful effect (*red dotted* “T”)

A few factors including genetic makeup (occurrence of wild type/mutated VGSC) and insecticide concentration might interpret our observations regarding pyrethroid resistance modulation. Mosquitoes expressing only wild type (WT) VGSC, or co-expressing WT and mutated VGSCs are susceptible when exposed to standard pyrethroid concentrations, indicating that the mutated allele has a recessive effect (reviewed in [42]). Consistently, in the absence of WT channels (i.e. mutated VGSC are exclusively expressed), resistance to pyrethroids is detected [18, 53]. This raises the question: how could RNAi targeting the modified VGSC restore pyrethroid susceptibility of resistant mosquitoes? We propose a model in which gene silencing shifts the VGSC: pyrethroid ratio. The consequence of this ratio alteration is higher local insecticide concentration per target site that eventually affects modified channel function. In this scenario the reduced activity or affinity of the pyrethroid to the modified VGSC is compensated by higher insecticide availability, leading to increased binding, which in turn leads to increased toxicity (Fig. 7). Further supporting this mode of action, pyrethroid-induced mortality of resistant RJ mosquitoes is elevated in a dose-dependent manner (Fig. 3a).

It has been shown that enhanced detoxification activity is associated with increased expression of related metabolic components including the P450 enzymes [48, 54]. Interestingly, we detected enhanced VGSC expression in the resistant RJ strain compared to the sensitive Rock strain. Although such target expression variation might be irrelevant to pyrethroid susceptibility, it is tempting to propose a potential effect. At low pyrethroid concentrations, overexpression of the wild type VGSC might shift the toxin: target ratio, leaving enough pyrethroid-free channels to stabilize the sodium concentration within axonic cells. Alternatively, overexpression of VGSC could potentially compensate fitness costs derived from newly evolved structural modifications; hence, preserving housekeeping properties while acquiring new traits. Lastly, a cooperative interaction between highly expressed toxin chelating alleles and wild type functional alleles would be important when novel target gene modifications evolve. Nevertheless, in all scenarios, knockdown of VGSC is predicted to compromise pyrethroid insensitivity, as demonstrated by the RNAi experiments in this study.

The aim of this study was to provide a feasible RNAi solution that can be applied on wild pyrethroid resistant mosquito populations in the near future. To achieve this, high dsRNA efficacy at economic quantities is required. It is recognized that the sodium channel transcript variability governs its functional diversity including the emergence of insecticide resistance [55, 56]. Therefore, to maximize the RNAi effect we tiled a number of overlapping dsRNA constructs that together target about half of the VGSC transcript variants annotated in this work (Fig. 5a). Despite having only 45 % of the gene covered, following the I-score prediction of siRNA regions we were able to cover 15 out of 22 siRNA regions pointed out using the I-Score Designer software (Additional file 9). This strategy provided a refined dsRNA trigger (tile-6) that increased mortality with a three-fold decrease in dsRNA amounts compared to the primary VGSC dsRNA construct. Since tile-6 is not homologous to a unique VGSC sequence and not predicted to be enriched with high numbers of active siRNAs (Additional file 9), its relatively high potency is not well understood. We postulate that local secondary structure of target transcripts plays a role in dsRNA-derived siRNAs’ efficiency. It is acknowledged that in field applications dsRNA has to be packaged to preserve its stability, quantity and delivery. Further addressing such requirements, similar increased susceptibility to the adulticide effect occurred when dsRNA was complexed by a polyamine reagent (see Additional file 10), indicating that naked dsRNA is not crucial for efficient dsRNA delivery to *Ae. aegypti* larvae*.*

RNAi has been applied to modulate mosquito gene expression in vivo mostly in the adult stage. A few studies reported on environmental dsRNA uptake (soaking or ingestion) and systemic RNAi effect in the larval stage [35, 57]. Lately, Whyard et al. showed that treating larvae with dsRNA corresponding to testis-specific genes resulted in male sterility phenotype in adults [37]. However, it has not been established molecularly whether RNAi that is induced in the larval stage can persist until adulthood. Our findings demonstrate an active prolonged cross-developmental stage, gene silencing that is effective for at least 35 days post-dsRNA introduction. The detection of VGSC-specific siRNAs (Fig. 4) coupled with VGSC silencing in adults (treated with dsRNA-VGSC as larvae) (Fig. 2e and 3b) is evidence that a sufficient amount of dsRNA was taken up by the larva and that active RNAi was sustained into adulthood. Interestingly, dsRNA-mediated VGSC knockdown had no apparent deleterious effect on treated larvae and adults in the absence of the pyrethroid insecticide. This implies that the VGSC gene is not a suitable RNAi target to induce mortality in *Ae. aegypti* possibly due to insufficient knockdown levels*.*

**Fig. 8:**
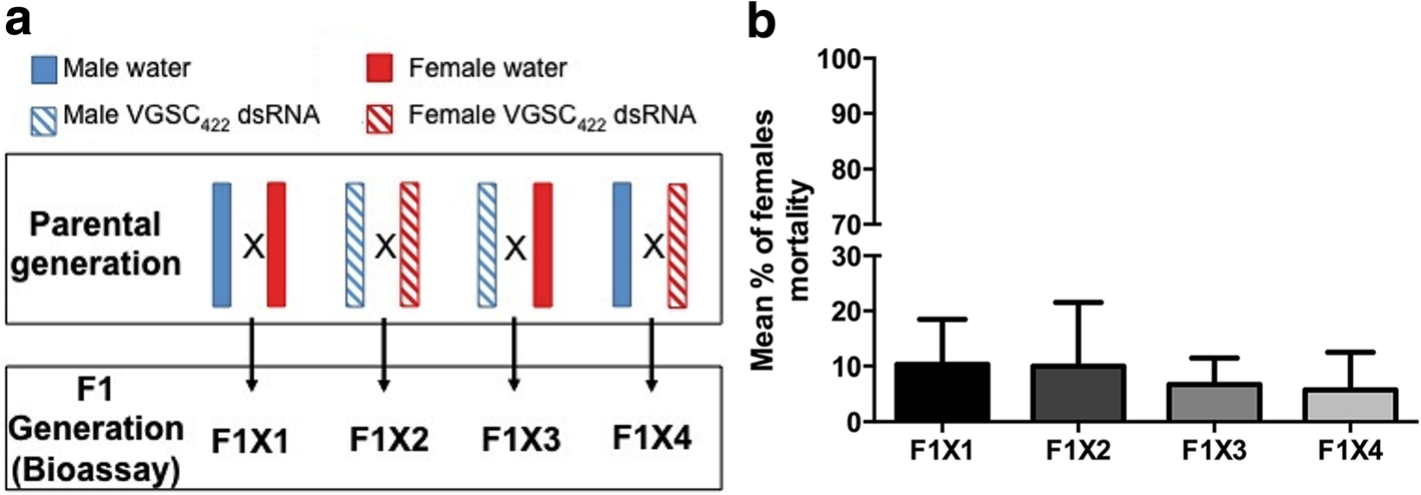
Effect of dsRNA-VGSC_422_ treatment in F0 on untreated F1 generation. Five day-old males and females previously soaked with dsRNA-VGSC_422_ or only water (F0; Parental Generation) were mated as represented in the scheme (left). The F1 generation resulting from indicated mating was subjected to a bioassay with 0.5 μg/ml of deltamethrin and the mortality is shown (right graph)

In *Caenorhabditis elegans*, the dsRNA-triggered gene silencing is amplified by RNA-dependent RNA polymerases as evidenced by secondary siRNAs, and a mobile RNAi signal could systemically spread to the germ line, resulting in transgenerational gene silencing [58]. Our data indicated that despite the potency of the dsRNA treatment, no secondary amplification of the silencing signal occurred (Fig. 4). Moreover, our crossing experiments showed that dsRNA-mediated gene silencing is not passed on to the subsequent generation in *Ae. aegypti* (Fig. 8). This could be explained by the degradation over time of the dsRNA trigger due to the absence of RNA-dependent RNA polymerase activity in insects including mosquitoes [50, 51]. It is also consistent with the dose-dependent persistence over time of dsRNA previously reported in adult mosquitoes [36].

## Conclusions

In conclusion, this work has demonstrated a novel strategy for the control of pyrethroid insecticide-resistant, disease vectoring mosquitoes. We applied dsRNA to *Ae. aegypti* larvae and showed long-lasting gene silencing that affects adults. The RNAi system was directed to impair resistance to pyrethroids by knocking down the well-established pyrethroid target, the voltage-gated sodium channel. Solving the pyrethroid resistance problem is of great importance as pyrethroids are the only class of insecticide approved to treat bed-nets [52]. Moreover, the approach reported in this study has the potential to reduce the environmental use of chemicals with broad-spectrum insecticidal activity such as pyrethroids. It is proposed that the dsRNA-triggered long term RNAi could be applied into various complementary strategies to control mosquitoes at different developmental stages. Finally, it should be noted that based on the procedure reported herein, we are working on additional avenues to achieve disease vector control.

## Abbreviations

dsRNA, double-stranded RNA; IRS, indoor residual spraying; PgP, *P-glycoprotein* gene; RISC, RNA-induced silencing complex; RJ, Rio de Janeiro; RNAi, RNA interference; Rock, Rockefeller strain; RPM, reads per million; siRNAs, small interfering RNAs; VGSC, voltage-gate sodium channel; WT, wild type; μg, microgram

## Additional files

Additional file 1: Table showing the list of primers used in Real-time PCR (qPCR) and in dsRNA production. (PDF 197 kb) Additional file 2: Artificial random dsRNA sequence with no perfect homology greater than 19 bp with the *Ae. aegypti* genome (taxid: 7159). (PDF 34 kb) Additional file 3: Table showing the list of primers used to amplify sodium channel gene mutations in *Ae. aegypti*. (PDF 162 kb) Additional file 4: Table showing the frequency of the 1016 and 1534 mutations in the sodium channel gene of the Rio de Janeiro strain of *A. aegypti*. (PDF 86 kb) Additional file 5: Soaking *Ae. aegypti* larvae (RJ strain) with specific dsRNA results in P-glycoprotein and CYP9J26 gene silencing during the larval and/or adult stages. (A-C) Larvae were soaked in water (untreated) or in dsRNA corresponding to the (A) P-glycoprotein (PgP) and P450 enzymes (B) CYP9J26 and (C) CYP9J32. Gene expression levels were determined by qRT-PCR and performed on untreated or dsRNA-treated (0.5 μg/μl) *Ae. aegypti* RJ larvae. Data are shown as the mean ± standard deviation of four biological replicates. Statistics: One-way Anova, followed by Tukey’s multiple comparison test. (D-F) Larvae were soaked as described above, and grown to the adult stage. Individual females were then analyzed for gene expression as described above. Data are shown as the mean ± SD of 7 females, and the experiment was performed 3 times. Statistics: Multiple *t*-test. **P* < 0.01. (TIFF 141 kb) Additional file 6: Table showing the list of all the transcript variants of VGSC available in the literature. (PDF 168 kb) Additional file 7: Screening for dsRNA sequence targeting VGSC gene. Mortality percentage of *Ae. aegypti* RJ adult females after 30 min exposure to pyrethroid deltamethrin. The adult females were treated at the 3^rd^ larval stage by different dsRNA-VGSC tiles (0.17 μg/ul). The graph shows the mean ± standard deviation of three replicates. Statistics: One-way Anova, followed by Tukey’s multiple comparison test.* *P* < 0.01. (TIF 144 kb) Additional file 8: DsRNA-VGSC_T6_ application at the larval stage does not affect vitality and behavior of treated mosquitoes. (A-B) Percentage of immature mortality (larvae and/or pupae) after dsRNA treatment (dsRNA-Random or dsRNA-VGSC_T6_) in the Rockefeller strain (A) and Rio de Janeiro strain (B). Data are shown as the mean ± standard deviation of three treatment replicates from two independent experiments. (C-D) Sex ratio in the Rockefeller (C) and Rio de Janeiro (D) strains after dsRNA treatment with dsRNA-Random or dsRNA-VGSC_T6._ Data are shown as the mean ± standard deviation of three treatment replicates of two independent experiments. (E-F) Percentage of blood fed females in the Rockefeller (E) and Rio de Janeiro (F) strains after dsRNA treatment (dsRNA-Random or dsRNA-VGSC_T6_) in the larval stage. Data are shown as the mean ± standard deviation of three treatment replicates of two independent experiments. (G-H) Number of eggs per female in the Rockefeller (G) and Rio de Janeiro (H) strains after dsRNA treatment (dsRNA-Random or dsRNA-VGSC_T6_) in the larval stage. Data are shown as the mean ± standard deviation of three treatment replicates of two independent experiments. (I-J) Hatching rate in the Rockefeller (I) and Rio de Janeiro (J) strains after treatment with dsRNA-Random or dsRNA-VGSC_T6_ in the larval stage. Data are shown as the mean ± standard deviation of three treatment replicates of two independent experiments. (TIFF 297 kb) Additional file 9: Table showing the list and hotspot sequences for siRNA in VGSC gene, using the I-Score Designer software. (PDF 218 kb) Additional file 10: Alternative dsRNA delivery using solid food. Groups of 100 3^th^ instar-larvae from *Ae. aegypti* RJ strain were fed with three plugs of 300 μl each containing 300 μg of VGSC_422_ dsRNA complexed with PEI and dissolved in 2 % of pre-melted agarose and food solution. Then, larvae were reared until adult stage and exposed to 0.5 μg/ml of deltamethrin, as described in A. The percentage of mortality after 30 min is shown. Statistics: Two-way Anova, followed by Sidak’s multiple comparison test. **P* < 0.05. (TIFF 241 kb)
