# Supplementary material for: Larval application of sodium channel homologous dsRNA restores pyrethroid insecticide susceptibility in a resistant adult mosquito population
Source: Parasit Vectors. 2016 Jul 14;9:397. doi: 10.1186/s13071-016-1634-y (PMC4946210; doi:10.1186/s13071-016-1634-y)
Supplement: Additional file 1: — Table showing the list of primers used in Real-time PCR (qPCR) and in dsRNA production. (PDF 197 kb) [file 13071_2016_1634_MOESM1_ESM.pdf]

Additional file 1 - Table. List of primers used in Real-time PCR (qPCR) and in dsRNA production

Gene	Primer Sequence (5’-3’)*	Position in the gene	Applica-tion	Access number
**Tubulin**	*Forward*: CTGCCACCTTCATCGGTAAC<br>*Reverse*: TGTTCGGAGATACGCTTGAAC	-	qPCR	XM_001655975.1
**Sodium channel (VGSC)**	*Forward*: CGGAGGCGTTCAATCGGATA<br>*Reverse*: TGGACATACCCCTTTGCCTG	-	qPCR	KC107440.1
**VGSC Tile 1**	*Forward*: TAATACGACTCACTATAGGGAGA ACTGGTTTTGGTCGGAAGAA<br>*Reverse*: TAATACGACTCACTATAGGGAGA TGAAAACAGTGGATGTACTAAAATAT	148-465	dsRNA production	KC107440.1
**VGSC Tile 2**	*Forward:* TAATACGACTCACTATAGGGAGA ATCCTATACGTCGCGTAGCT<br>*Reverse:* TAATACGACTCACTATAGGGAGA ACATTGTTAAAATTATCACATC	416-837	dsRNA production	KC107440.1
**VGSC Tile 3**	*Forward:* TAATACGACTCACTATAGGGAGA TCATAGAGTCCGTTAAGAATCTC<br>*Reverse:* TAATACGACTCACTATAGGGAGA AGACGAAAGGCAGATAAGAATG	788-1145	dsRNA production	KC107440.1
**VGSC Tile 4**	*Forward:* TAATACGACTCACTATAGGGAGA TATACAAGTTTCGATACTTTCGGA<br>*Reverse:* TAATACGACTCACTATAGGGAGA TGCCCTTCTCCTGGTTCAC	1096-1495	dsRNA production	KC107440.1
**VGSC Tile 5**	*Forward:* TAATACGACTCACTATAGGGAGA GGACTTTTCCTGCCACAGCT<br>*Reverse:* TAATACGACTCACTATAGGGAGA GATGATTGCACCATTTTCCTC	1446-1782	dsRNA production	KC107440.1
**VGSC Tile 6**	*Forward:* TAATACGACTCACTATAGGGAGA ATGACTCGAACGCGGTCAC<br>*Reverse*: TAATACGACTCACTATAGGGAGA ATATCGTTTAACACCATCACGT	1733-2132	dsRNA production	KC107440.1
**VGSC Tile 7**	*Forward*: TAATACGACTCACTATAGGGAGA CAAACACAAACCGTAGTAGATATG<br>*Reverse*: TAATACGACTCACTATAGGGAGA TCGCAATCAGCTTCATCGTT	2083-2506	dsRNA production	KC107440.1
**VGSC Tile 8**	*Forward:* TAATACGACTCACTATAGGGAGA TTTTTTCACGGCGACCTTCG<br>*Reverse:* TAATACGACTCACTATAGGGAGA CGATG TAGTTCTTGCCGAACA	2457-2794	dsRNA production	KC107440.1
**Cytocrome p450 (CYP9J26)**	*Forward:*TAATACGACTCACTATAGGGAGA CTTCAACGACTTCGTCCGATCA<br>*Reverse:* TAATACGACTCACTATAGGGAGA GTCCACCTTGATTCCAAAAGCACAC	-	dsRNA production	JF924909.1<br>XM_001649047.2
**Cytocrome p450 (CYP9J32)**	*Forward:* TAATACGACTCACTATAGGGAGA TTCAACGATTTCATCCAGACCA<br>*Reverse:* TAATACGACTCACTATAGGGAGA GTAATCCACGTAGCCCGATTC	-	dsRNA production	XM_001653404.2

*The first 23 nucleotides in 5’ position of all primers used in dsRNA production refers to T7 promoter sequence.
