# Supplementary material for: Larval application of sodium channel homologous dsRNA restores pyrethroid insecticide susceptibility in a resistant adult mosquito population
Source: Parasit Vectors. 2016 Jul 14;9:397. doi: 10.1186/s13071-016-1634-y (PMC4946210; doi:10.1186/s13071-016-1634-y)
Supplement: Additional file 2: — Artificial random dsRNA sequence with no perfect homology greater than 19 bp with the Ae. aegypti genome (taxid: 7159). (PDF 34 kb) [file 13071_2016_1634_MOESM2_ESM.pdf]

Additional file 8 – Artificial random dsRNA sequence (5’ → 3’)

GTAAAACGACGGCCAGTGATGAGTCTGGGTGGAGCGCGCCCCATTTATAC
CGTGAGTAGGGTCGACCAAGAACCGCAAGATGCGTCGGTGTACAAATAAT
TGTCAACAGACCGTCGTGTTTTGAAAATGGTACCAGCATCTTCGGGCGGT
CTCAATCAAGCATGGATTACGGTTGAACTAATACGTATACTTTGCACGGG
TTCACTGCGGTCCGTTCAGAGTCGACCAAGGACACAATCGAGCTCCCATC
TGTATGCTCGACTAACTTGTACCCAACCCCCGGAGCTTGGCAGCTCCTGGG
GTATCATGGAGCCTCTGGTTCATCCCGTGGGATATCAAGCCATGGTCATA
GCTGTT
