# Supplementary material for: Larval application of sodium channel homologous dsRNA restores pyrethroid insecticide susceptibility in a resistant adult mosquito population
Source: Parasit Vectors. 2016 Jul 14;9:397. doi: 10.1186/s13071-016-1634-y (PMC4946210; doi:10.1186/s13071-016-1634-y)
Supplement: Additional file 3: — Table showing the list of primers used to amplify sodium channel gene mutations in Ae. aegypti. (PDF 162 kb) [file 13071_2016_1634_MOESM3_ESM.pdf]

Additional file 2 - Table. Primers used to amplify sodium channel gene mutations in *Ae. aegypti*

Mutation	Primer	Primer Sequence (5'-3')
**1016**	Val+ Forward	##ACAAATTGTTTCCCACCCGCACCGG
	Ile Forward	#ACAAATTGTTTCCCACCCGCACTGA
	1016 Reverse	GGATGAACCGAAATTGGACAAAAGC
**1534**	Phe Forward	#TCTACTTTGTGTTCTTCATCATATT
	Cys Forward	##TCTACTTTGTGTTCTTCATCATGTG
	1534 Reverse	TCTGCTCGTTGAAGTTGTCGAT
	##long 5'-tail	GCGGGCAGGGCGGCGGGGGCGGGGCC
	#short 5'- tail	GCGGGC
