# Supplementary material for: Larval application of sodium channel homologous dsRNA restores pyrethroid insecticide susceptibility in a resistant adult mosquito population
Source: Parasit Vectors. 2016 Jul 14;9:397. doi: 10.1186/s13071-016-1634-y (PMC4946210; doi:10.1186/s13071-016-1634-y)
Supplement: Additional file 4: — Table showing the frequency of the 1016 and 1534 mutations in the sodium channel gene of the Rio de Janeiro strain of A. aegypti. (PDF 86 kb) [file 13071_2016_1634_MOESM4_ESM.pdf]

Additional file 3. Table. Frequency of the 1016 and 1534 mutations in the sodium channel gene of the Rio de Janeiro strain of *A. aegypti*

**Genotype Frequency**				
**Phenotype**	**Mutation #1016**		**Mutation #1534**	
Susceptible	Val/Val	0.31	Phe/Phe	0.05
	Val/Ile	0.20	Phe/Cys	0.35
Resistant	Ile/Ile	0.49	Cys/Cys	0.60
**Allelic Frequency**				
Wild	Val	0.41	Phe	0.23
Mutant	Ile	0.59	Cys	0.77
