# Supplementary figures and images for: Larval application of sodium channel homologous dsRNA restores pyrethroid insecticide susceptibility in a resistant adult mosquito population
Source: Parasit Vectors. 2016 Jul 14;9:397. doi: 10.1186/s13071-016-1634-y (PMC4946210; doi:10.1186/s13071-016-1634-y)

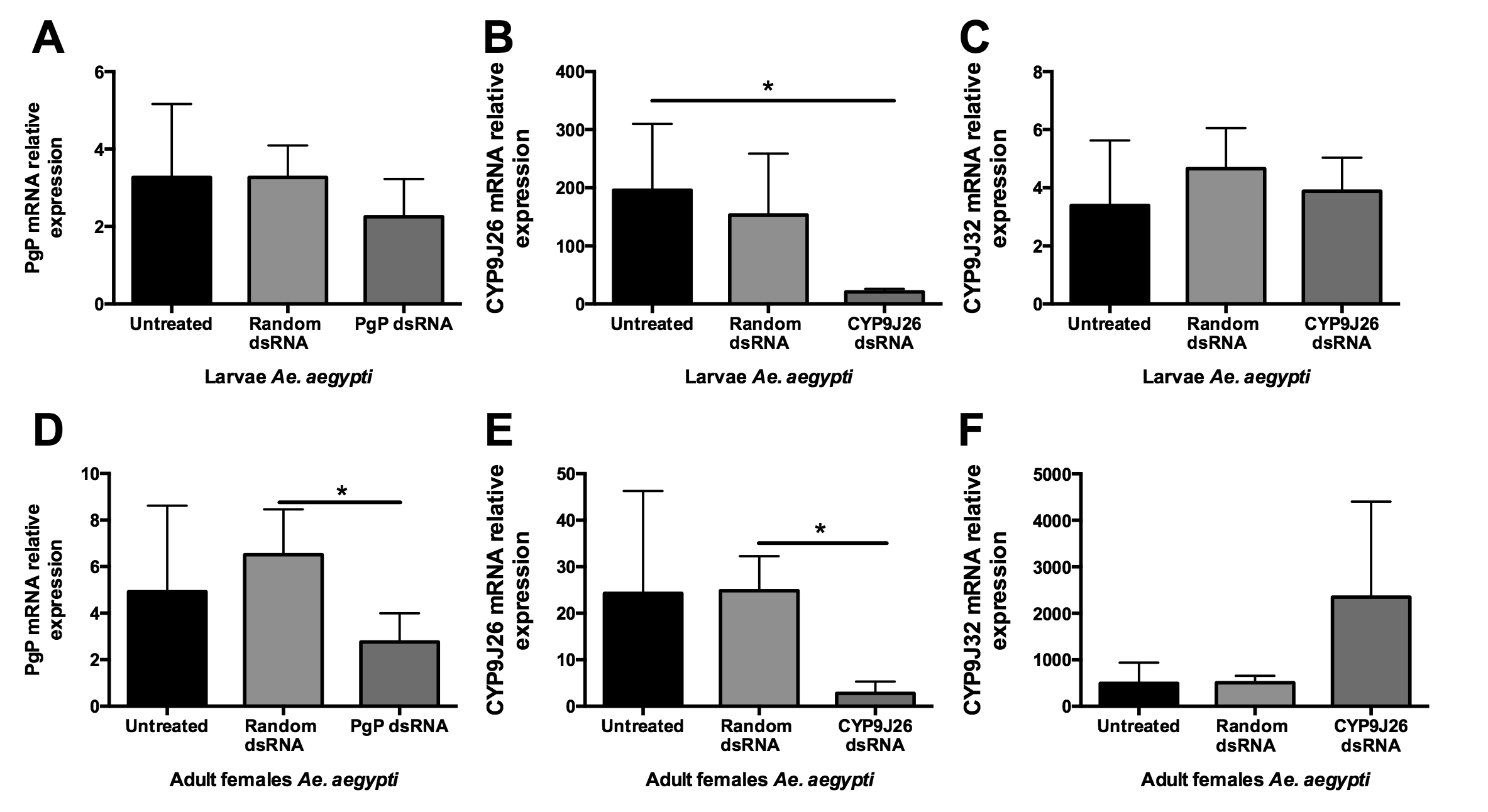

Supplement: Additional file 5: — Soaking Ae. aegypti larvae (RJ strain) with specific dsRNA results in P-glycoprotein and CYP9J26 gene silencing during the larval and/or adult stages. (A-C) Larvae were soaked in water (untreated) or in dsRNA corresponding to the (A) P-glycoprotein (PgP) and P450 enzymes (B) CYP9J26 and (C) CYP9J32. Gene expression levels were determined by qRT-PCR and performed on untreated or dsRNA-treated (0.5 μg/μl) Ae. aegypti RJ larvae. Data are shown as the mean ± standard deviation of four biological replicates. Statistics: One-way Anova, followed by Tukey’s multiple comparison test. (D-F) Larvae were soaked as described above, and grown to the adult stage. Individual females were then analyzed for gene expression as described above. Data are shown as the mean ± SD of 7 females, and the experiment was performed 3 times. Statistics: Multiple t-test. *P < 0.01. (TIFF 141 kb) [file 13071_2016_1634_MOESM5_ESM.tiff]

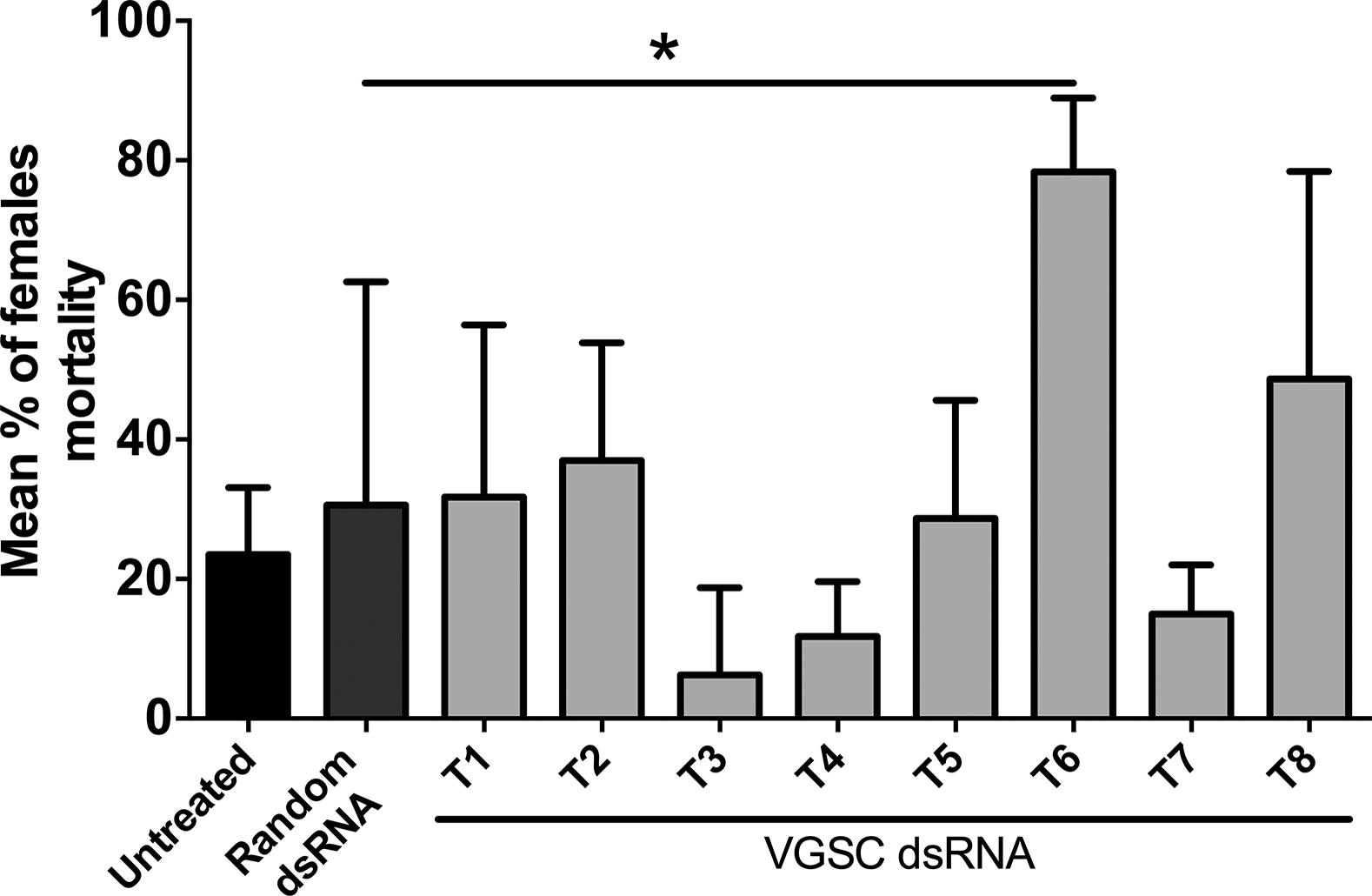

Supplement: Additional file 7: — Screening for dsRNA sequence targeting VGSC gene. Mortality percentage of Ae. aegypti RJ adult females after 30 min exposure to pyrethroid deltamethrin. The adult females were treated at the 3rd larval stage by different dsRNA-VGSC tiles (0.17 μg/ul). The graph shows the mean ± standard deviation of three replicates. Statistics: One-way Anova, followed by Tukey’s multiple comparison test.* P < 0.01. (TIF 144 kb) [file 13071_2016_1634_MOESM7_ESM.tif]

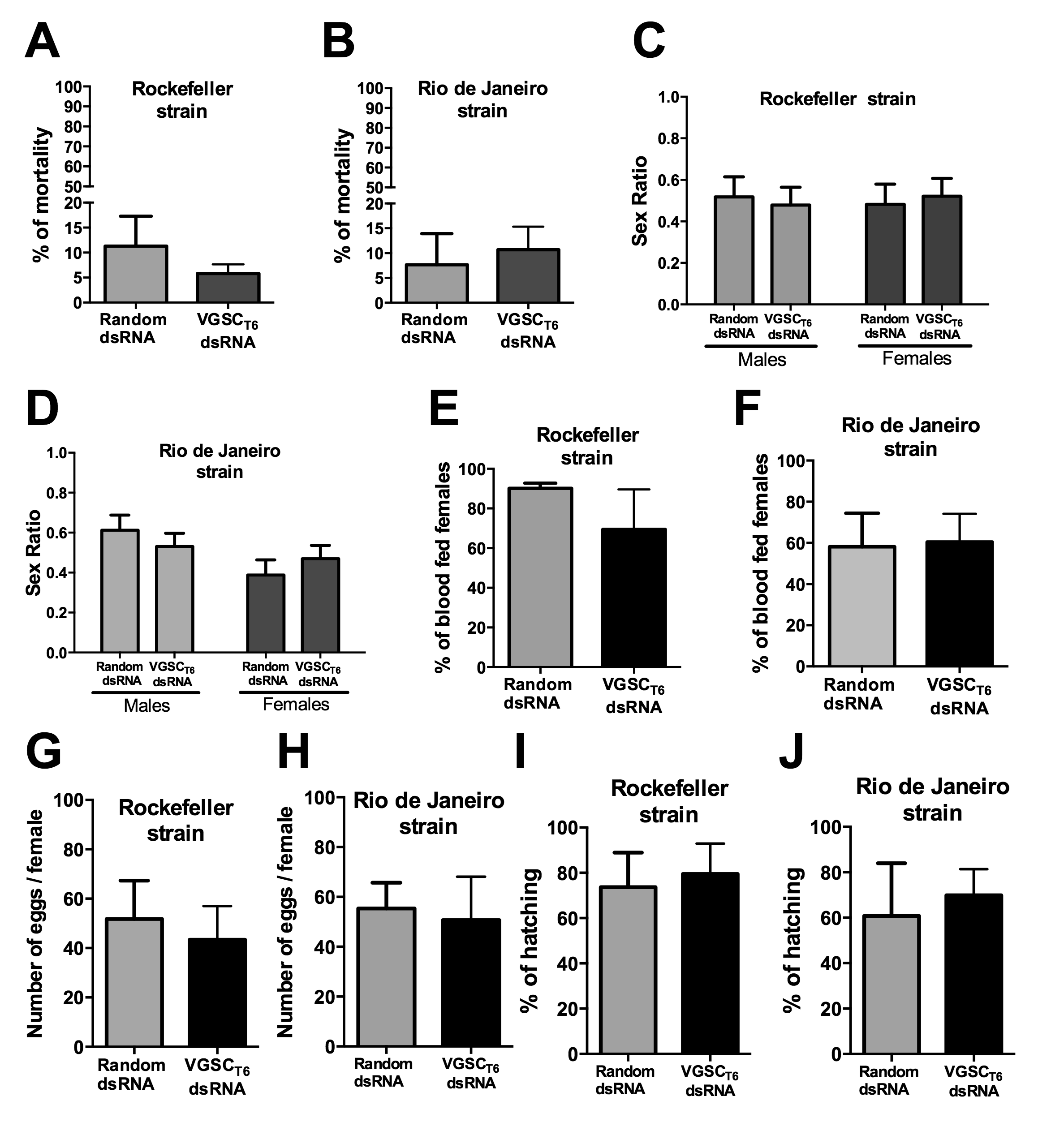

Supplement: Additional file 8: — DsRNA-VGSCT6 application at the larval stage does not affect vitality and behavior of treated mosquitoes. (A-B) Percentage of immature mortality (larvae and/or pupae) after dsRNA treatment (dsRNA-Random or dsRNA-VGSCT6) in the Rockefeller strain (A) and Rio de Janeiro strain (B). Data are shown as the mean ± standard deviation of three treatment replicates from two independent experiments. (C-D) Sex ratio in the Rockefeller (C) and Rio de Janeiro (D) strains after dsRNA treatment with dsRNA-Random or dsRNA-VGSCT6. Data are shown as the mean ± standard deviation of three treatment replicates of two independent experiments. (E-F) Percentage of blood fed females in the Rockefeller (E) and Rio de Janeiro (F) strains after dsRNA treatment (dsRNA-Random or dsRNA-VGSCT6) in the larval stage. Data are shown as the mean ± standard deviation of three treatment replicates of two independent experiments. (G-H) Number of eggs per female in the Rockefeller (G) and Rio de Janeiro (H) strains after dsRNA treatment (dsRNA-Random or dsRNA-VGSCT6) in the larval stage. Data are shown as the mean ± standard deviation of three treatment replicates of two independent experiments. (I-J) Hatching rate in the Rockefeller (I) and Rio de Janeiro (J) strains after treatment with dsRNA-Random or dsRNA-VGSCT6 in the larval stage. Data are shown as the mean ± standard deviation of three treatment replicates of two independent experiments. (TIFF 297 kb) [file 13071_2016_1634_MOESM8_ESM.tiff]

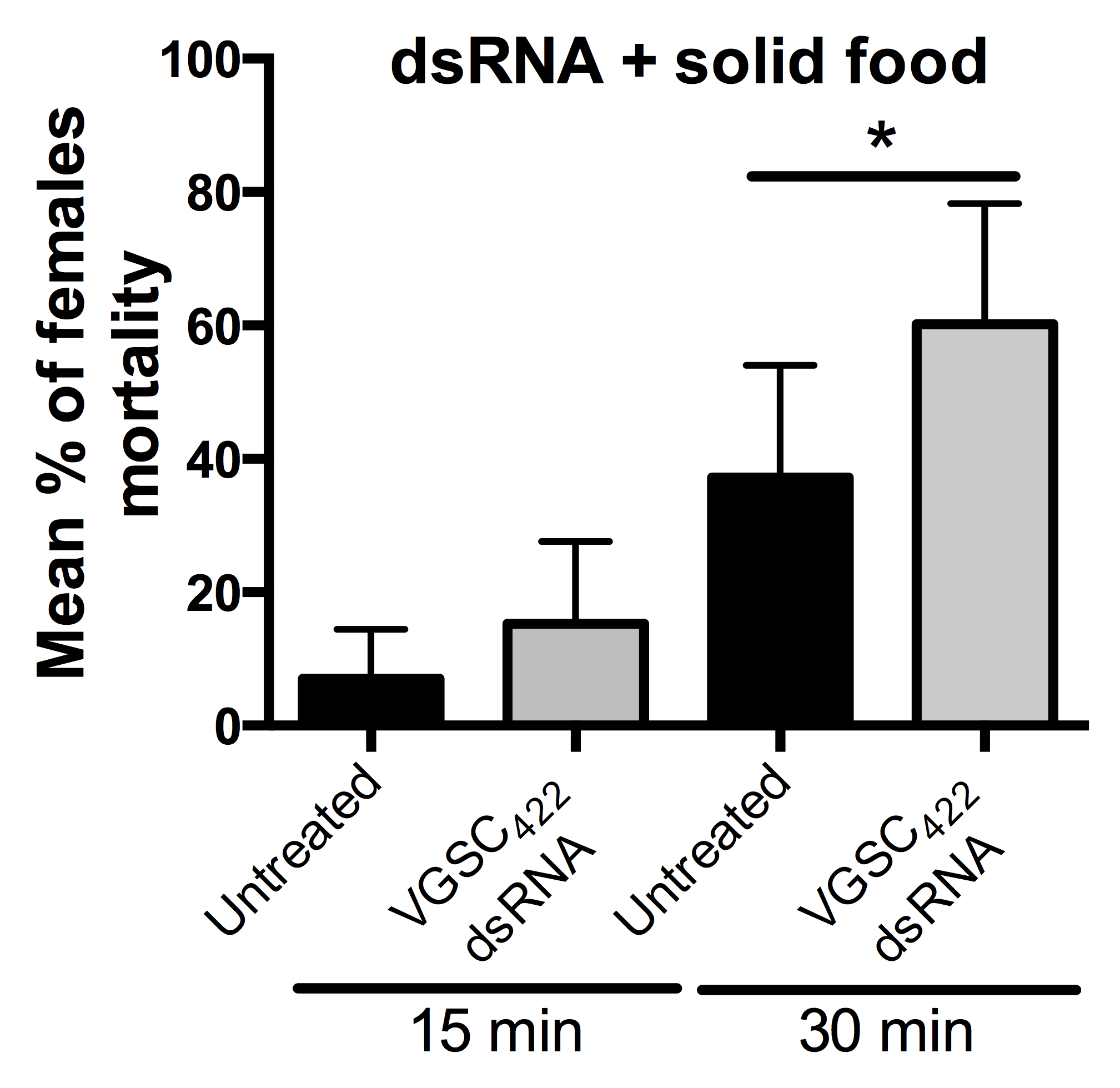

Supplement: Additional file 10: — Alternative dsRNA delivery using solid food. Groups of 100 3th instar-larvae from Ae. aegypti RJ strain were fed with three plugs of 300 μl each containing 300 μg of VGSC422 dsRNA complexed with PEI and dissolved in 2 % of pre-melted agarose and food solution. Then, larvae were reared until adult stage and exposed to 0.5 μg/ml of deltamethrin, as described in A. The percentage of mortality after 30 min is shown. Statistics: Two-way Anova, followed by Sidak’s multiple comparison test. *P < 0.05. (TIFF 241 kb) [file 13071_2016_1634_MOESM10_ESM.tiff]
