# Supplementary material for: Larval application of sodium channel homologous dsRNA restores pyrethroid insecticide susceptibility in a resistant adult mosquito population
Source: Parasit Vectors. 2016 Jul 14;9:397. doi: 10.1186/s13071-016-1634-y (PMC4946210; doi:10.1186/s13071-016-1634-y)
Supplement: Additional file 6: — Table showing the list of all the transcript variants of VGSC available in the literature. (PDF 168 kb) [file 13071_2016_1634_MOESM6_ESM.pdf]

Sodium channel gene variants

length	exon#	KC107440|Waco	AY663378|clone_AEL2	AY663377|clone_AER2	EU792890|clone_pK33	EU784647|clone_PN9	AY663385.1|partial	EU399180|NS	EU399179|Bora	AAEL006019-RD	AAEL006019-RA	AAEL006019-RB	AAEL006019-RC	XM_001649431.1|AAEL004612-RA	AAEL008297-RA	EU399181|Per-R	AB909019|SMK	XM_001663390.1|AAEL013277-RA
147	1	1-127	-	-	-	-	-	1-147	1-147	-	-	-	-	1-127	-	1-147	1-127	
33	2(j)	128-160	-	-	-	-	-	148-180	148-180	-	-	-	-	128-160	1-13	148-180	128-160	
156	3	161-317	-	-	1-56	1-56	1-128	181-337	181-337	-	-	-	-	161-317	14-170	181-337	161-317	
206	4	318-523	-	-	57-262	57-262	129-334	338-543	338-543	-	-	-	-	318-346	171-376	338-543	318-523	
129	5	524-652	-	-	263-391	263-391	335-463	544-672	544-672	-	-	-	-	-	377-505	544-672	524-652	
92	6	653-744	-	-	392-483	392-483	464-555	673-764	673-764	1-73	1-73	1-73	1-73	-	506-597	673-764	653-744	
213	7	745-957	-	-	484-696	484-696	556-768	765-977	765-977	74-286	74-286	74-286	74-286	-	598-810	765-977	745-957	
61	8	958-1018	-	-	697-757	697-757	769-829	978-1038	978-1038	287-347	287-347	287-347	287-347	-	811-917	978-1038	958-1018	
145	9	1019-1163	1-17	-	758-902	758-902	830-974	1039-1183	1039-1183	348-492	348-492	348-492	348-492	-	918-1058	1039-1183	1019-1163	
165	10	1164-1328	18-182	-	903-1067	903-1067	975-1139	1184-1348	1184-1348	493-657	493-657	493-657	493-657	-	1059-1223	1184-1348	1164-1328	
276	11_i	1329-1604	183-458	-	1068-1343	1068-1343	1140-1415	1349-1624	1349-1624	658-876	658-933	658-933	658-933	-	1224-1499	1349-1624	1329-1604	
21	12(n)	1605-1623	459-478	-	1344-1363	1344-1363	1416-1435	1625-1644	1625-1644	-	934-954	934-952	934-953	-	1500-1519	1625-1645	1605-1624	
63	13(a)	-	479-521	-	1364-1406	1364-1406	1436-1478	1645-1687	1645-1687	877-921	955-1017	-	954-996	-	1520-1562	1646-1708	1625-1667	
184	14_m_14	1624-1788	522-705	-	1407-1590	1407-1590	1479-1662	1688-1871	1688-1871	922-1087	1018-1183	953-1117	997-1180	-	1563-1746	1709-1892	1668-1851	
203	15	1789-1991	706-908	-	1591-1793	1591-1793	1663-1865	1872-2074	1872-2074	1088-1272	1184-1368	1118-1320	1181-1383	-	1747-1949	1893-2095	1852-2054	
100	16	1992-2091	909-1008	-	1794-1893	1794-1893	1866-1965	2075-2174	2075-2174	1273-1372	1369-1468	1321-1420	1384-1483	-	1950-2049	2096-2195	2055-2154	
69	17_b_17	2092-2160	1009-1077	-	1894-1962	1894-1962	1966-2034	2175-2243	2175-2243	1373-1441	1469-1537	1421-1489	1484-1552	-	2050-2118	2196-2264	2155-2223	
278	18	2161-2438	1078-1331	-	1963-2216	1963-2240	2035-2312	2244-2521	2244-2521	1442-1719	1538-1791	1490-1743	1553-1830	-	2119-2372	2265-2542	2224-2477	
174	19	2439-2612	1332-1505	-	2217-2390	2241-2414	2313-2486	2522-2695	2522-2695	1720-1893	1792-1965	1744-1917	1831-2004	-	2373-2546	2543-2716	2478-2651	421-437 (106-122)
163	20(d)	2613-2775	1506-1590	-	2391-2553	2415-2577	2487-2649	2696-2858	2696-2858	1894-2056	1996-2128	1918-2080	2005-2167	-	2547-2709	2717-2879	2652-2814	592-614 (92-114)
188	21	2776-2963	-	1-119	2554-2741	2578-2765	2650-2837	2859-3046	2859-3046	2057-2244	2129-2316	2081-2268	2168-2355	-	2710-2748	2880-3067	2815-3002	763-802 (106-145)
247	22_e	2964-3191	-	120-342	2742-2968	2766-3012	2838-3065	3047-3293	3047-3293	2245-2471	2317-2539	2269-2495	2356-2583	-	-	3068-3314	3003-3229	
212	f_f'_23	3192-3383	-	343-509	2969-3131	3013-3194	3066-3257	3294-3505	3294-3505	2472-2634	2540-2706	2496-2658	2584-2775	-	-	3315-3526	3230-3392	
220	h_24	3384-3603	-	510-651	3132-3273	3195-3414	3258-3477	3506-3725	3506-3725	2635-2776	2707-2848	2659-2800	2776-2995	-	-	3527-3746	3393-3612	
266	25	3604-3869	-	652-917	3274-3539	3415-3680	3478-3743	3726-3991	3726-3991	2777-3042	2849-3114	2801-3066	2996-3261	-	-	3747-4012	3613-3878	
174	26	3870-4043	-	918-1091	3540-3713	3681-3854	3744-3917	3992-4165	3992-4165	3043-3216	3115-3288	3067-3240	3262-3435	-	-	4013 - 4186	3879-4052	
123	27(l)	4044-4166	-	1092-1214	3714-3836	3855-3977	3918-4040	4166-4288	4166-4288	3217-3339	3289-3411	3241-3363	3436-3558	-	-	4187-4309	4053-4175	
123	28	4167-4289	-	1215-1337	3837-3959	3978-4100	4041-4163	4289-4411	4289-4411	3340-3462	3412-3534	3364-3486	3559-3681	-	-	4310-4432	4176-4298	
195	29	4290-4484	-	1338-1532	3960-4154	4101-4295	4164-4358	4412-4606	4412-4606	3463-3657	3535-3729	3487-3681	3682-3876	-	-	4433-4627	4299-4493	1792-1802 (130-140)
246	30	4485-4730	-	1533-1728	4155-4400	4296-4541	4359-4604	4607-4852	4607-4852	3658-3903	3730-3975	3682-3927	3877-4122	-	-	4628-4873	4494-4739	1931-2010 (63-142), 2018-2028 (206-216)
271	31	4731-5001	-	-	4401-4671	4542-4812	4605-4875	4853-5123	4853-5123	3904-4174	3976-4246	3928-4198	4123-4393	-	-	4874-5144	4740-5010	2338-2354 (232-248)
305	32	5002-5306	-	-	4672-4976	4813-5117	4876-5180	5124-5428	5124-5428	4175-4479	4247-4551	4199-4503	4394-4698	-	-	5145-5449	5011-5315	2563-2573 (186-196)
>993	33	5307-6321	-	-	4977-5991	5118-6132	5181-6317	5429-6554	5429-6554	4480-5575	4552-5647	4504-5599	4699-4794	-	-	5450-6580	5316-6330	2722-2733 (40-51), 2875-2897 (193-215)
