# Supplementary material for: Larval application of sodium channel homologous dsRNA restores pyrethroid insecticide susceptibility in a resistant adult mosquito population
Source: Parasit Vectors. 2016 Jul 14;9:397. doi: 10.1186/s13071-016-1634-y (PMC4946210; doi:10.1186/s13071-016-1634-y)
Supplement: Additional file 9: — Table showing the list and hotspot sequences for siRNA in VGSC gene, using the I-Score Designer software. (PDF 218 kb) [file 13071_2016_1634_MOESM9_ESM.pdf]

Additional file 6 – Table. Definition of the best hotspot sequences for siRNA using the I-Score Designer software

| Hotspot position within the gene according to I-Score Designer* | Hotspot sequence | Number of appearances of the hotspot according to I-Score Designer* | Match of hotspots inside tiling regions |
|---|---|---|---|
| 151 | GGTTTTGGTCGGAAGAAAA | 2 | tile 1 |
| 157 | GGTCGGAAGAAAAAGAAAA | 1 | tile 1 |
| 162 | GAAGAAAAAGAAAAAAGAA | 1 | tile 1 |
| 429 | CGTAGCTATTTATATTTTA | 1 | tile 1, tile 2 |
| 457 | CTGTTTTCATTTTTTATAA | 1 | tile 1, tile 2 |
| 566 | CGTTCGAATCAGCTGTAAA | 2 | tile 2 |
| 810 | CAGAGATGTGATAATTTTA | 2 | tile 2, tile 3 |
| 1069 | GGTTATGGAGATAATCCAA | 3 | tile 3 |
| 1240 | GGTTCGTTCTACCTTGTAA | 1 | tile 4 |
| 1463 | GCTACGAGCTGTTCGTGAA | 1 | tile 4,tile 5 |
| 1619 | GCGCGTTTACGATACGTAA | 1 | tile 5 |
| 2001 | GCGAGACTTTGATATGTCA | 1 | tile 6 |
| 2477 | CGATAGAAGCAACGATGAA | 1 | tile 7, tile 8 |
| 2631 | GCTTCGAGTGTTCAAGCTA | 1 | tile 8 |
| 2636 | GAGTGTTCAAGCTAGCGAA | 1 | tile 8 |
| 3134 | CGCTCAAGTTCGTGAAAAA | 2 | - |
| 3329 | GGATGGAGTTCACGATACA | 1 | - |
| 3483 | CGATGATACTGCCAGTATA | 1 | - |
| 3962 | CGGTGATATTTTTTCTAGA | 1 | - |
| 4006 | GGTTTTCGAGTTTACTTTA | 1 | - |
| 5267 | GGCTGGACGATGTGTACAA | 1 | - |
| 6135 | GGCTAACTTAGGTATAGTA | 1 | - |

*Hotspot definition using I-Score Designer software according to prediction scores of Best i-Scores, Best s-Biopredsi and Best DSIR

Reference I-Score Designer:

Ichihara M, Murakumo Y, Masuda A, Matsuura T, Asai N, Jijiwa M, Ishida M, Shinmi J, Yatsuya H, Qiao S, Takahashi M, Ohno K. Thermodynamic instability of siRNA duplex is a prerequisite for dependable prediction of siRNA activities. Nucleic Acids Res 35:e123, 2007
